# What have we learned from brucellosis in the mouse model?

**DOI:** 10.1186/1297-9716-43-29

**Published:** 2012-04-13

**Authors:** María-Jesús Grilló, José María Blasco, Jean Pierre Gorvel, Ignacio Moriyón, Edgardo Moreno

**Affiliations:** 1 Instituto de Agrobiotecnología, CSIC-UPNA-Gobierno de Navarra, Pamplona, Spain; 2 Centro de Investigación y Tecnología Agroalimentaria (CITA) de Aragón, Zaragoza, Spain; 3 Centre d'Immunologie de Marseille-Luminy, Aix Marseille Université, Faculté de Sciences de Luminy, Luminy, France; 4 Institut National de la Santé et de la Recherche Médicale U631, Marseille, France; 5 Centre National de la Recherche Scientifique UMR6102, Marseille, France; 6 Departamento de Microbiología y Parasitología, Universidad de Navarra, Pamplona, Spain; 7 Instituto de Salud Tropical, Universidad de Navarra, Pamplona, Spain; 8 Programa de Investigación en Enfermedades Tropicales, Escuela de Medicina Veterinaria, Universidad Nacional, Heredia, Costa Rica; 9 Instituto Clodomiro Picado, Facultad de Microbiología, Universidad de Costa Rica, San José, Costa Rica

## Abstract

Brucellosis is a zoonosis caused by Brucella species. Brucellosis research in natural hosts is often precluded by practical, economical and ethical reasons and mice are widely used. However, mice are not natural Brucella hosts and the course of murine brucellosis depends on bacterial strain virulence, dose and inoculation route as well as breed, genetic background, age, sex and physiological statu of mice. Therefore, meaningful experiments require a definition of these variables. Brucella spleen replication profiles are highly reproducible and course in four phases: i), onset or spleen colonization (first 48 h); ii), acute phase, from the third day to the time when bacteria reach maximal numbers; iii), chronic steady phase, where bacterial numbers plateaus; and iv), chronic declining phase, during which brucellae are eliminated. This pattern displays clear physiopathological signs and is sensitive to small virulence variations, making possible to assess attenuation when fully virulent bacteria are used as controls. Similarly, immunity studies using mice with known defects are possible. Mutations affecting INF-γ, TLR9, Myd88, Tγδ and TNF-β favor Brucella replication; whereas IL-1β, IL-18, TLR4, TLR5, TLR2, NOD1, NOD2, GM-CSF, IL/17r, Rip2, TRIF, NK or Nramp1 deficiencies have no noticeable effects. Splenomegaly development is also useful: it correlates with IFN-γ and IL-12 levels and with Brucella strain virulence. The genetic background is also important: Brucella-resistant mice (C57BL) yield lower splenic bacterial replication and less splenomegaly than susceptible breeds. When inoculum is increased, a saturating dose above which bacterial numbers per organ do not augment, is reached. Unlike many gram-negative bacteria, lethal doses are large (≥ 10^8^ bacteria/mouse) and normally higher than the saturating dose. Persistence is a useful virulence/attenuation index and is used in vaccine (Residual Virulence) quality control. Vaccine candidates are also often tested in mice by determining splenic Brucella numbers after challenging with appropriate virulent brucellae doses at precise post-vaccination times. Since most live or killed Brucella vaccines provide some protection in mice, controls immunized with reference vaccines (S19 or Rev1) are critical. Finally, mice have been successfully used to evaluate brucellosis therapies. It is concluded that, when used properly, the mouse is a valuable brucellosis model.

## Table of content

Introduction

Infection models

The *Brucella* stains: replication patterns and related effects

Route of the infection

Infective dose

The mouse

Resistant and susceptible mouse strains

Mutant and knockout mice

Age and sex

Transmission

Physiopathology

Onset of the infection

Acute phase

Acute phase in pregnant mice

Chronic steady phase

Chronic declining phase

Vaccination

Superinfection and antigen therapy

Passive transfer and immunomodulation

Antibiotic treatment

Concluding remarks

Endnotes

Competing interests

Authors’ contributions

Acknowledgments

References

## Introduction

The genus *Brucella* comprises at least eight species named according to their preferred mammal hosts. *Brucella melitensisBrucella abortus* and *Brucella suis* are the most economically important species and they preferentially infect goats and sheep, bovines and swine, respectively [1]. Livestock is the source of human infections, and brucellosis is a severe disease that affects a considerable number of people in the world [1]. These bacteria cause long lasting chronic infections, mainly colonizing the reticuloendothelial system and reproductive organs [2,3], replicating in the internal milieu of trophoblasts, macrophages and dendritic cells [4]. Although able to multiply in life-less media, *Brucella* organisms are better described as facultative extracellular intracellular parasites [5].

For many years the pathophysiology of brucellosis was investigated in humans and natural hosts [3,6-9]. However, experimentation in ruminants, humans and primates has economical and ethical concerns or is precluded for practical reasons. Consequently, small laboratory animals are frequently employed as models in brucellosis research. One of the first experimental models was the chicken embryo [10]. Although this model was useful for evaluating the intracellular multiplication of *Brucella*, it does not differentiate virulent from attenuated strains. The rabbit has been used in protocols designed to study *Brucella* toxicity and hypersensitivity, mainly because of its susceptibility to bacterial endotoxins and toxins [11]. Due to practical reasons related to size, management and cost, the rabbit has never been widely used as a model in brucellosis, although it is used to produce antibodies against *Brucella* antigens [12]. Owing to their high susceptibility to *Brucella* infections and similarities in reproducing human pathology, the guinea pig was extensively used as an experimental animal [13]. These rodents reproduce the pulmonary, hepatic, spleen and genital lesions and the hypersensitivity reactions observed in humans, and match the different phases of the infection caused by *Brucella* in natural hosts, including abortion [13-15]. Thus, the guinea pig is one of the best models and it is still used for some immunological and vaccine studies [16,17]. However, when large numbers of animals are necessary, guinea pigs become impractical for the same reasons as rabbits. Other laboratory rodents such as rats, hamsters and gerbils have been used sporadically [13].

The mouse (*Mus musculus*) has been the most widely used brucellosis model. Mice were first used by Holth in 1911 for *Brucella* vaccine testing. Thereafter, mice were used for the etiological confirmation of samples from infected animals, to test virulence and for the evaluation of the pathological lesions (see [18,19]). The results in mice are not immediately applicable and transferable to humans or to the target animal species. However, the uncovering of a significant phenotype in mice using an appropriate protocol gives useful information. With the arrival of inbred, mutant, knockout and transgenic mice and the understanding of their biology and immunology [20,21], this rodent has become the standard model for brucellosis research.

In this work we have reviewed the models of infection by O-polysaccharide containing (smooth) *Brucella* species and strains in mice. Although we have focused on in vivo assays, we occasionally refer to ex vivo studies in cells when they are relevant for understanding the biology of smooth brucellae in mice. For reasons related to the extent of the document, we have avoided reviewing the infection models induced by the rough brucellae. For a better understanding on the biological behavior of the rough strains in mice, we suggest the work of González et al. [22].

## Infection models

The outcome of the infection in mice depends on the virulence and dose of the *Brucella* strain, the route of inoculation, and on the breed, genetic background, age, sex and physiological status of the mice. Since most studies in mice are devoted to investigate virulence, pathogenicity, immunology and vaccine properties, it is critical to include control groups inoculated with appropriate reference *Brucella* strains [23,24]. In the following sections, we discuss the importance of these factors.

### The *Brucella* strains: replication patterns and related effects

The affinity of some *Brucella* species for a particular mammal host is well-known [1]. It is also notorious that *B. melitensis* and some biovars of *B. suis* infect humans more frequently and cause a more severe disease than *B. abortus*[1]; though, these infectivity and virulence patterns do not always reproduce in mice. For instance, in a comparative study it was found that the order of virulence in mice was *B. melitensis* H38 (biovar 1) > *B. abortus* 2308 (biovar 1) > *B. suis* 1330 (biovar 1) [25]. Moreover, there are differences in the pathological behavior among biovars and strains of some virulent species, and the virulence demonstrated for their target hosts does not necessarily parallels that observed in mice [25,26]. The appraisal of these issues becomes more complicated when different *Brucella* strains are simultaneously inoculated for comparative purposes. In addition to the eight recognized *Brucella* species and several biotypes (and more in the “waiting list”), a large number of bacterial mutants and constructs have been developed and tested in the murine system. In general, three different categories of *Brucella* strains may be distinguished in relation to pathogenicity, ability to multiply and persist in mice: virulent, attenuated and avirulent.

Although most organs of the reticuloendothelial system may become infected (depending upon the bacterial dose), the spleen and liver are the most conspicuously infected organs in mice. In these organs, virulent smooth brucellae (e.g. *B. abortus* 2308 and 544, *B. melitensis* 16 M and H38, and *B. suis* 1330) show a very reproducible pattern that is clearly different from that of the attenuated vaccines and the non-virulent brucellae. In the initial phases, the numbers of CFU/organ are similar in the liver and in the spleen, but the number of CFU/g of organ is lower in liver (one to two logarithms). In subsequent phases, the CFU are consistently lower in the liver [27,28] and virulent brucellae are completely cleared from this organ beyond 3–4 weeks post-infection (pi) [29]. This pattern is somewhat different from that observed in spleens (see Physiopathology). In splenectomized mice, the liver becomes rapidly colonized [15]. It seems therefore, that the higher *Brucella* colonization of the spleen during the chronic phases guards the liver from a profound inflammation [15]. Due to this, liver is seldom used for estimating the number of CFU, and the spleen is the preferred target organ to study *Brucella* infections in mice.

**Figure 1:**
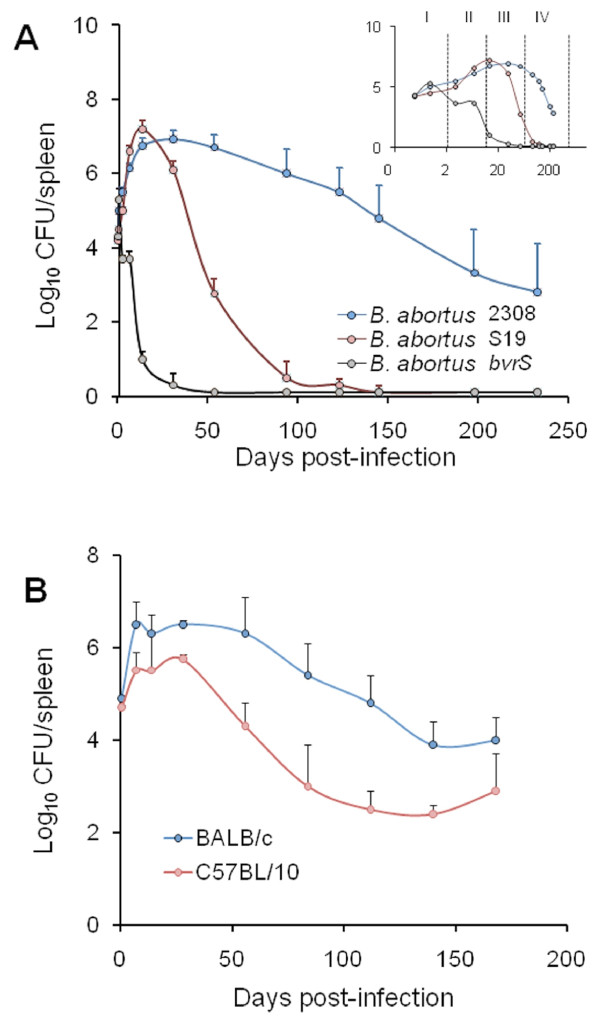
**Replication profiles of* B. abortus* in mice spleens.** (**A**) Spleen replication curves of virulent (2308 strain), vaccine-attenuated (S19 strain) and non-virulent (*bvrS* mutant) *B. abortus* strains during 36 weeks after inoculation in CD-1 mice. The abscissas axis (time after infection) of the inserted panel is expressed in logarithmic scale, to allow a better understanding of the initial phases of infection. The different phases of the infection (I, onset of infection; II, acute phase; III, chronic steady phase; IV, chronic declining phase) are depicted by the discontinuous vertical lines. (**B**) Spleen replication curves of virulent *B. abortus* 2308 strain in susceptible BALB/c and resistant C57BL/10 mice (adapted from [35], with permission).

The enumeration of bacteria in spleen (expressed as the mean ± SD of individual Log_10_ CFU/spleen or Log_10_ CFU/g of spleen; Figure 1) provides highly reproducible replication profiles. The lapse of the different infection phases (Figure 1) may vary depending upon the inoculation protocol. Nevertheless, at the optimal dose of infection (see below) a consistent replication profile for the reference *Brucella* strains is maintained within a certain range [29]. In the understanding that infection with virulent *Brucella* (at standard doses, see below) is a continuous process and that delimitations are not clear cut, this replication profile can be divided into four different phases (Figure 1A): i) the onset of the infection, marked by colonization during the first 48 h pi; ii) the acute phase, extending from the 3^rd^ day to the time when CFU reach their maximum, generally between weeks 2 and 3; iii) the chronic steady phase, that corresponds to the CFU plateau, commonly lasting 8–11 weeks; and iv) the chronic declining phase, at which there is a slow elimination of the bacteria that may last beyond 36 weeks. The span of these phases may vary depending upon the bacterial dose, route, mouse strain and age [30]. Generally, experiments in mice are not prolonged more than 3–4 months and, therefore, data on the *Brucella* persistence in these animals after this period are scarce. It has been documented that *Brucella* organisms may still be recovered from the spleen and lymph nodes of mice after 6 months of infection [25,31], suggesting that virulent *Brucella* might remain in mice for life. Although some quantitative variations have been observed, the spleen replication patterns of virulent strains follow similar kinetics [24,25]. Fully avirulent strains (e.g. *B. abortus bvrS* mutant; Figure 1A) are unable to multiply or persist, regardless of the dose. In contrast, attenuated strains (e.g. S19 and Rev1) can multiply at the levels of the virulent strains at the early phases (Figure 1A) but persist for shorter times, even when inoculated at large doses (e.g. 10^8^ CFU/animal) [31]. The degree to which attenuated bacteria are able to persist is the basis of the recommended Residual Virulence quality control of anti-*Brucella* vaccines [16]. It is expressed as the Recovery Time 50 (RT50), i.e. the time (in weeks) at which the bacterium is eliminated from the spleens in half of vaccinated mice. Used in this way, the murine model has demonstrated its usefulness to detect batches of poor immunogenic reference vaccines [32-34].

The course of *Brucella* infections induced by attenuated strains, such as vaccines S19 and Rev1, or non-virulent mutants, such as VirB or BvrS [36,37], is radically shortened and modified (Figure 1A). Similarly, the replication profiles in knockout or mutant mice may vary according to the defect displayed by the corresponding mouse strain (Table 1). In the case of vaccine S19 (which shows a conspicuous Residual Virulence), the replication kinetics in the spleen follows a rapid increase that peaks between weeks 1 and 3, and then decreases steadily at approximately one logarithm per week [23,24]. Nonetheless, this vaccine may still be recovered from spleens 8 to 12 weeks pi. The replication profile of vaccine Rev1 shows some differences with respect to that of S19. In general, Rev1 does not display the rapid increase demonstrated by S19, declines more slowly, and is still present after 8 to 12 weeks [33]. As stated above, non-virulent strains do not multiply or increase in the initial phases of the infection, and then decline very fast (Figure 1A) being eliminated from the spleen and the liver between the 2^nd^ and 7^th^ week [37,38].

**Table 1:** **Replication and persistence of smooth virulent*Brucella*in mutant and knockout mice strains**

Test mouse strain	Phenotypic defect	Reference mouse strain	*Brucella* spleen CFU increase and persistence relative to mouse reference strain^a^		References
			Early before 14 days	Late after 15 days	
CBA/H	Inbred no specific defect	BALB/c	↓	↑	[39]
CBA/H	Inbred no specific defect	C57BL/10	↑	↑	[39]
CBA/NJ	Inbred no specific defect	BALB/c	↔	↔	[40]
DBA2	Inbred no specific defect	C57BL/6	↑	↑	[41,42]
CD-1	Outbreed Swiss mice	BALB/c	↔	↔	[40]
BALB/c	Inbred no specific defect	C3H/HeN	↔	↔	[40]
BALB/c	Inbred no specific defect	C57BL/6	↑	↑	[39]
BALB/c	Inbred no specific defect	C57BL/10	↑	↑	[39]
BALB/c	Inbred no specific defect	C.CB (Nramp1r)	↓	↔	[43]
C57BL/10	Inbred no specific defect	B10Br	↔	↔	[39]
C57BL/6 (TLR9)	CpG motifs recognition	C57BL/6	↑	↑	[44,45]
BALB/c (TLR9)	CpG motifs recognition	BALB/c	↔	↔	[46]
BALB/c (TLR4)	LPS detection	BALB/c	↔	↔	[46]
BALB/c (TLR4)	LPS detection	BALB/c	↑	↔	[44]
C3H/HeJ (TLR4)	LPS detection	C3H/HePas	↑	↑	[47]
C3H/HeJ (TLR4)	LPS detection	C3H/HeAu	↔	↔	[48]
C3H/HeJ (TLR4)	LPS detection	C3H/Heb	↔	↔	[41,42]
C3H/HeJ (TLR4)	LPS detection	C3H/HeN	↔	↔	[40,49]
C3H/HeJ (TLR4)	LPS detection	BALB/c	↔	↔	[40]
C57BL/6 (TLR4)	LPS detection	C57BL/6	↔	↔	[50]
C57BL/6 (TLR2)	Lipoproteins detection and peptidoglycan detection	C57BL/6	↔	↔	[47,50]
BALB/c (TLR2)	Lipoproteins detection and peptidoglycan detection	BALB/c	↔	↑	[46]
BALB/c (TLR2)	Lipoproteins detection and peptidoglycan detection	BALB/c	↔	↔	[44]
C57BL/6 (TLR2/4)	LPS and lipoproteins detection	C57BL/6	↔	↔	[50]
BALB/c (TLR2/4)	LPS and lipoproteins detection	BALB/c	↑	↔	[44]
C57BL/6 (NOD1)	muramyl peptides meso-diaminopimelic acid	C57BL/6	↔	ND	[51]
C57BL/6 (NOD2)	muramyl dipeptides	C57BL/6	↔	ND	[51]
C57BL/6 (Myd88)	Low proinflammatory response	C57BL/6	↑	↑	[44,45,50]
C57BL/6 (TRIF)	Low proinflammatory response	C57BL/6	↔	↔	[44]
C57BL/6 (TRIF)	Low proinflammatory response	129 Sv/Ev	ND	↔	[44]
C57BL/10 (IRAK-4)	Low proinflammatory response	C57BL/6	↑	↔	[52]
C57BL/6 (Rip2)	NOD adaptor protein	C57BL/6	↔	ND	[51]
C57BL/6 (*gp91*^*phox*^)	Low respiratory burst in phagocytes	C57BL/6	↔	↑	[53]
C57BL/6 (iNOS)	Low respiratory burst in phagocyte	C57BL/6	↑	↑	[44]
C57BL/6 (iNOS)	Low respiratory burst in phagocytes	C57BL/6	↔	↑	[53]
C57BL/6 (IL-12p40)	Early differentiation of Th1 cells	C57BL/6	↔	↑	[53]
DBA/2j xC57BL/6 (iNOS/IL-12p40)	Early differentiation of Th1 cells and low respiratory burst	DBA/2j xC57BL/6	↔	↑	[53]
DBA/2 J (ICSBP)	Deficient in IL-12p40 and low respiratory burst	C57BL/6	↑	↑	[53]
C57BL/6 xDBA/2 J (iNOS/ICSBP)	Deficient in IL-12p40 and low respiratory burst	C57BL/6 xDBA/2 J	↑	↑	[53]
C57BL/6 (IRF-2)	Deficient in NK cells and dysregulation of IL-12p40	C57BL/6	↓	↔	[53]
C57BL/6 (*igh6*)	Affects B cells	C57BL/6	↔	↔/↓	[54]
C57BL/6 (*igh6*)	Affects B cells	C57BL/6	↓	↓	[55]
BALB/c (*jh*)	Affects B cells	BALB/c	↓	↓	[55]
C57BL/6 (r*ag-1*)	Affects B and T cells	C57BL/6	↔	↔	[54]
C57BL/6 (Cd4)	Affects CD4 T cells	C57BL/6	↔	↓	[54]
C57BL/6 (Aβ)	Affects CD4 T cells	C57BL/6	↓	↔	[56]
C57BL/6 (Aβ)	Affects CD4 T cells	C57BL/6	↔	↔	[55]
C57BL/6 (*Pfp*)	Affects Restriction of Tc mediated killing	C57BL/6	↑	↔	[57]
C57BL/6 (*β2m*)	Affects CD8 T cells	C57BL/6	↑	↑	[56]
C57BL/6 (*β2m*)	Affects CD8 T cells	C57BL/6	↔	↔	[54]
C57BL/6 (*β2m*)	Affects CD8 T cells	C57BL/6	↑	↔	[57]
C57BL/6 (*β2m*)	Affects CD8 T cells	C57BL/6	↔	↓	[55]
C57BL/6 (IL12/*β2m*)	Affects CD8 T cells and early differentiation of Th1 cells	C57BL/6	↑	↑	[58]
C57BL/6 (*nu/nu*)	Lack thymus derived T cells	C57BL/6	↓	↑	[59]
BALB/c (*ifng*)	Absence of IFN-γ	BALB/c	↔	↑ Dead	[57]
C57BL/6 (*ifng*)	Absence of IFN-γ production	C57BL/6	↑	↑	[44,54,58]
C57BL/6 (*ifng*)	Absence of IFN-γ production	C57BL/6	↔	↑ Dead	[57]
C57BL/6 (*ifng*)	Absence of IFN-γ production	C57BL/6	↔	↑	[55]
C57BL/6 (*ifng*)	Absence of IFN-γ production	C57BL/6	↑	ND	[60]
C57BL/6 (IRF-1)	CD8 T and NK cells dysregulation of IL-12p40 low respiratory burst	C57BL/6	↑	↑ Dead	[53]
BALB/c (*infar1)*	More susceptible to viral Infections. Affects NK	BALB/c	↔	↔	[61]
C57BL/6 (INFαβR)	More susceptible to viral Infections. Affects NK	129 Sv/Ev	ND	↓	[62]
C57BL/6 (TCRδ)	Absence of Tγδ cells	C57BL/6	↑	↔	[60]
C57BL/6 (GM-CSF)	Higher lung infection Defective alveolar macrophages	C57BL/6	↔	↔	[60]
C57BL/6 (IL-17Rα)	Defective in PMN recruitment and PMN activity	C57BL/6	↔	ND	[60]
C57BL/6 (IL-1β)	Low proinflammatory response	C57BL/6	↔	↔	[50]
C57BL/6 (IL-18)	Early activation of NK and Th1 cells	C57BL/6	↔	↔	[50]
C57BL/6 (IL-1βx IL-18)	Low proinflammatory response and recruitment of NK and Th1 cells	C57BL/6	↔	↔	[50]
C57BL/10 (TNF-α)	Low proinflammatory response	C57BL/10	↑	ND	[63]

*Brucella* susceptibility order: CBA/H>CBA/NJ=CD-1=BALB/c=C3H/HeN=C3H/HeJ>C57BL/10=C57BL/6≥B10Br.

^a^ (↑) Significantly higher numbers of CFU in spleen than in controls; (↓) significantly lower numbers of CFU in spleen than in controls; (↔) no significant number of CFU in spleen in relation to the controls; ND, not done.

**Figure 2:**
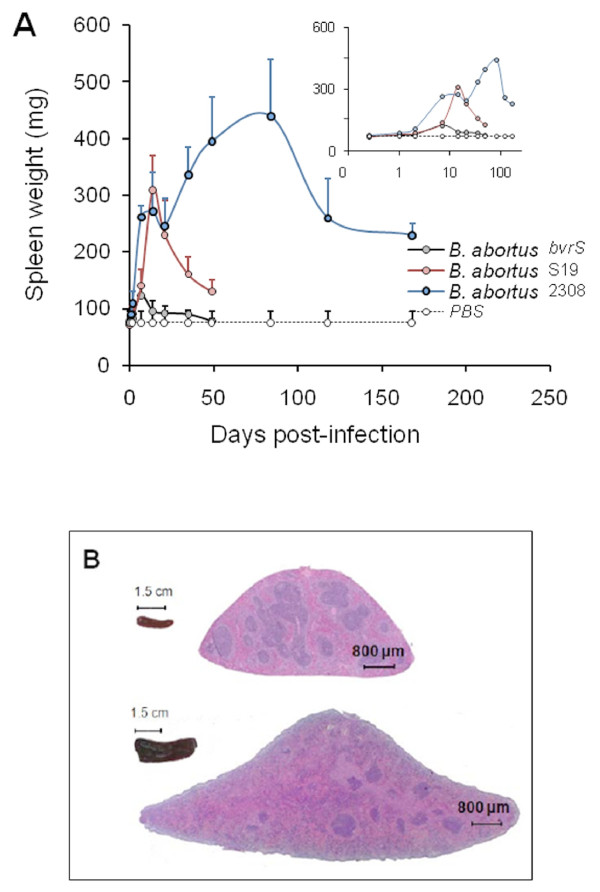
**Spleen inflammation in mice infected with *B. abortus*.** (**A**) Evolution of the average of spleen weights (as an indicator of inflammation) of CD-1 mice infected with virulent (2308 strain), vaccine-attenuated (S19 strain) and non-virulent (*bvrS* mutant) *B. abortus* strains during 25 weeks after inoculation. The abscissas axis (time after infection) of the inserted panel is expressed in logarithmic scale to allow a better understanding of spleen inflammation at the initial phases of infection. (**B**) Comparison of spleen size (left) and histological transversal sections stained with hematoxylin-eosine of normal spleen (upper panel) and *B. abortus* 2308 infected spleen, after 8 weeks pi (lower panel). Notice that the proportion between white pulp/red pulp in the normal spleen is close to 1/1, while in the infected spleen is close to 1/4. The histological section of normal mouse is from Dr Frank Voelker, Flagship Biosciences, with permission of Steve Pots Charting A New Course in Tissue Analysis [69].

Simultaneously with bacterial replication, there is swelling of the spleen (Figure 2) and liver as well as draining through lymphatics from the site of the infection (see Physiopathology). The enlargement of the spleen is characterized by a weight peak (e.g. ≥ 400 mg) evident from weeks 3 to 16 (Figure 2A). This enlargement is a consequence of inflammation [25] and it depends on the *Brucella* dose and virulence [64], as well as on the immune status and genetic background of the mice [50,53,65-67] (see Physiopathology). For example, *B. melitensis* H38 induces an intense splenic enlargement that generally surpasses that induced by other strains such as *B. melitensis* 16 M, *B. suis* 1330 or *B. abortus* 544. Attenuated S19 vaccine induces a characteristic peak of splenomegaly that occurs close to 2 weeks after inoculation and that, depending on the dose, may exceed that of *B. abortus* 2308 or 544. However, while in S19 infected mice the weight of the spleen rapidly decreases, the spleen of mice inoculated with virulent *Brucella* keeps increasing up to the end of the chronic steady phase (Figure 1B). Finally, killed *Brucella* or non-virulent strains (e.g. *B. abortus* BvrR/BvrS or VirB mutants) barely cause an increase in spleen size, even when injected in very large quantities (e.g. > 5 × 10^7^/mouse) [68]. This corresponds to a general phenomenon linked to the rapid removal and killing of non-virulent strains by professional phagocytes.

Quality control of the *Brucella* strains is of paramount importance. In addition to its tendency to dissociate into rough forms [1,70], smooth brucellae are prone to become attenuated upon storage at 4°C, after subculturing, or in old or prolonged cultures without showing appreciable biochemical or bacteriological modifications [32]. In vitro passages should be reduced to a minimum, and the original strain kept for comparison. To rule out attenuation as much as possible, it is recommended that the *Brucella* strain be expanded only once, and then frozen at −80°C or in liquid nitrogen until used. When expanded, these frozen stocks should maintain the expected parameters of the strain and pass at least one additional test in macrophages: non-opsonized smooth virulent brucellae multiply in non-activated macrophages (e.g. RAW 264.7, J774, human monocytes or bone marrow derived) and show a characteristic replication curve [66]. This profile often shows a 0.5 to 1.5 logarithm drop during the first 12 to 15 h, and then steadily levels up and increases 1 to 2 logs after 24 to 48 h. If the *Brucella* strain keeps a steady state after the critical period of 12 h (as it is the case of *B. abortus* S19) or drops quickly (like the avirulent VirB or BvrS-BvrR mutants), it is either attenuated or non-virulent. Alternatively, the macrophages may have been activated (e.g. endotoxin in the culture media), increasing their bactericidal abilities [66]. Once the *Brucella* strain has passed this test, the stored aliquots of the same stock shall be expanded and assayed in mice. Indeed, it may happen that some strains showing the adequate profile in macrophages fail to display a virulent profile in mice and are thus attenuated. This is the case of some mutants that, nevertheless, pass the multiplication test in macrophages [22,71]. One alternative procedure when hesitating about the quality of a *Brucella* strain of known virulence is to inoculate the bacterium into mice and then recover the organisms from the spleen after 2 to 3 weeks [37,72]. Once selection of the virulent phenotype has been ensured, the isolate has to be handled as described above, since some *Brucella* rough variants may arise in the spleens of infected mice [73].

Attenuated and non-virulent *Brucella* mutants present a particularly difficult problem because stocks of these strains in different laboratories frequently come from serial passages in vitro. This problem is exemplified by the differences in Residual Virulence found for various lots of vaccines [16,32,33]. A drawback of vaccine Rev1 is its tendency to dissociate from smooth to rough organisms. This event has a profound negative effect on the immunogenicity and Residual Virulence and efficacy, since rough bacteria are highly attenuated [32,33]. The simultaneous presence of large and small colonies (evidenced only after 4 days culture) is a common change observed in S19 vaccines stocks that may be related to virulence differences in mice [33]. Similarly, when making mutants by genetic manipulation, a number of passages (frequently in the presence of antibiotics) is necessary, and these steps bias the selection in favor of bacteria that grow preferentially in vitro and that may introduce further attenuation not associated with the specific genetic defect studied. Therefore, it is always recommended to balance these studies with the use of complemented mutant strains, even if this method does not necessarily restore the levels of the original in vivo behavior [37].

The above-described *Brucella* replication profiles can be expressed either as log_10_ CFU/organ or as log_10_ CFU/g of organ. However, in some cases the CFU/organ may give statistical significant differences, whereas the normalized CFU/g of organ values may erase this statistical significance. Expressing the CFU/gram, while correcting for individual variations due to inflammatory responses, eliminates information on the absolute bacterial numbers recovered, and then the CFU values are lower. It is thus better to express the CFU per organ. One concern about the latter method is that it assumes that all mice have closely similar spleen weights. If deemed necessary because there are large differences in spleen weights, it is possible to include the individual spleen weights and CFU/organ in two “dot” graphics. In most cases, however, the overall significance does not change.

### Route of the infection

Mice are commonly infected intraperitoneally (i.p.) or intravenously (i.v.) with doses ranging from 10^4^ to 10^7^ CFU/mouse in a volume of 0.2 to 0.05 mL. Both routes infect 100 % of the mice and induce similar levels of infection. However, the i.p. route is preferred: it is technically simpler, admits larger volumes and, therefore, it is less prone to inoculation errors. The i.p. route results in higher bacterial counts and faster colonization of the spleen than other organs [39,65,74]. On the other hand, the i.v. route (commonly in the tail vein) promotes a slightly faster and higher bacterial colonization of the liver in relation to the spleen during the first 10 days, an event that is reversed during the following 2 weeks and then maintained throughout the infection period, up to 120 days or more [29]. Smooth brucellae are quickly phagocytized by leukocytes following i.v. inoculation. The same phenomenon is observed after i.p. infection but with 1 to 3 h delay [14], suggesting that the bacteria promptly reach the blood via the thoracic duct and probably through the peritoneal capillary system as well. No matter if inoculated i.p. or i.v., bacteria are distributed throughout the reticuloendothelial system and placenta within the 1^st^ week, and depending on the dose, they can also be isolated in testes, joints and salivary glands [25,75,76]. The central nervous system of the adult normal mouse does not seem to be colonized when using these routes, even at high doses [76]. Although strict experimentation concerning the presence of the bacterium in the meninges has not been performed, the behavior of infected mice does not suggest brain infection. It is striking that in contrast to what has been reported in humans, dolphins and the fetuses of ungulates [77-79], there are no reports on neurobrucellosis in other juvenile or adult natural *Brucella* hosts, such as bovine, caprine or ovine. However, since domestic animals are most often culled upon evidence of the disease, this is an aspect that has not been studied in all its dimensions. Neurobrucellosis is quite an interesting syndrome because the hematoencephalic barrier imposes several unique conditions to the invading pathogen.

The subcutaneous (s.c.) inoculation, either in the back zone or in the footpad of mice results in lower levels of infection than the i.p. or i.v. routes [26,74,80,81]. This effect may be due to local recruitment of bacteria at the site of inoculation. The s.c. route in the back zone is recommended for quality control of vaccines [16]. Inoculation of *Brucella* suspensions (e.g. 10^5^ to 10^6^ CFU/mouse in 0.05 mL) into the footpad causes local inflammation; enhanced by the relatively large volume deposited in a small area that induces tissue destruction and the subsequent phagocytosis by resident leukocytes. After footpad injection a spreading of the *Brucella* infection takes place through the lymphatics, favoring the localization of the bacterium in the popliteal lymph node [75,82-84]. At about 1 h pi, brucellae are already detected in blood, spleen and liver, reaching a transient plateau in these organs 6 h later [83,84]. Then, they can extend to other organs [74]. The s.c. infection in the back zone (e.g. 10^5^–10^9^ CFU/mouse) follows a similar course as the footpad inoculation [74]. The s.c. inoculation of *B. abortus* or *B. melitensis* rarely induces pus. Nevertheless, depending on the bacterial dose and the volume injected, a local granuloma formed by mononuclear cells and neutrophils appears in the inoculation site after several days. Vaccine Rev1 can induce encapsulated local transient abscesses when inoculated by the s.c. route. However, this only happens at very high doses (≥ 10^8^ CFU) and abscesses are of benign nature being resolved in a few days/weeks [22], in parallel with what it is observed in sheep inoculated by s.c. route with the same strain [85].

The respiratory route of infection (through aerosols or intranasal) has been considered by some authors as the most natural route, and a source of laboratory accidents and of potential bio-terrorism attacks [86,87]. Leaving aside the inherent risks of this procedure, a precise assessment of the CFU inoculated is more inexact than when using other routes. The aerosol method requires estimating the bacterial inocula within a respiratory chamber by sacrificing a group of mice immediately after exposure to the aerosol [86,87]. Moreover, simultaneous infection trough conjunctival, nasal and oral mucosae cannot be excluded during aerosol exposure, with the ensuing problems in interpretation. The intranasal route of infection displays similar problems because mice are very good at sneezing, generating local aerosols. In any case, these routes of inoculation induce an immediate infection of the lungs, which is then distributed by blood to the reticuloendothelial system [86-88]. Early in lung infections, *Brucella* is present and replicates in alveolar macrophages. Then, bacteria are disseminated to the lung-draining mediastinal lymph nodes where they replicate in both migratory dendritic cells and migratory alveolar macrophages. These last phagocytic cells seem to be critical regulators of the early innate immune response within the lungs [4].

The oral route by gavage has been used in mice attempting to reproduce human or experimental goat [89] infection after ingestion of contaminated dairy products with *Brucella*[90-92]. However, the gastrointestinal route is inefficacious to infect humans [7,93] and mice [91] with *Brucella*. In addition to the challenges posed by the local microbiota, epithelial layers and specific local immune responses, the gastric acid pH and bile salts negatively affect bacteria that, like brucellae, hardly or not at all grow at pH below 5 and have outer membranes that are not barriers to hydrophobic substances [94]. The difficulties in infecting mice by this route may also be related to the robust local resistance associated with the presence of specialized Paneth cells in the intestinal tract. These cells are rich in bactericidal substances that, together with the strong acidic conditions, contribute to the gastrointestinal barrier in mice [21]. Moreover, there are some technical difficulties intrinsic to this route, and infection trough the oral mucosa and the “digestive” or “intestinal” infection are very different issues [3]. To achieve infection through gavage inoculation, very large numbers of *Brucella* (≥ 10^10^ CFU/mouse) have to be placed in the oro-esophageal cavity typically in 0.1 to 0.25 mL. Under these conditions, the plastic tubing or ball needles used in gavage are prone to deposit bacteria into the upper esophagus and in the oral cavity. Consequently, infections via both oral and throat mucous membranes are exceedingly difficult to rule out. Even with those large doses, only a low proportion of bacteria (1–2 %) translocate through gut cells, and are distributed in the reticuloendothelial organs. After 8 h, the bacteria may be recovered from the ileum, cecum and colon and to a lesser extent from the spleen, liver, mesenteric lymph nodes and Peyer´s patches. As expected, whereas in the intestine the numbers of bacteria decrease over time, the numbers in the spleen, liver and lymph nodes steadily increase during the first 2 weeks, indicating the high affinity of *Brucella* for the reticuloendothelial system. A proportion of mice do not show bacteria in the main target organs [91,92], consistent with the idea that *Brucella* infection through the gastrointestinal route is unfavorable. As an alternative to gavage, large numbers of *Brucella* have been injected into intestinal loops, favoring the internalization of significant bacterial numbers by ileal Peyer´s patches dendritic cells [95]. This procedure induces little inflammatory response.

### Infective dose

A myriad of bacterial doses have been used in experimental murine brucellosis. Indeed, it is essential to determine the dose retrospectively by plating aliquots of the inoculum [33,68]. To know how many live bacteria have been inoculated is critical because, whereas live brucellae predominantly induce a Th1 response [66], dead brucellae have a tendency to induce T-independent responses [96]. This precaution is even more relevant when different *Brucella* strains are compared.

*Brucella* hardly induces mortality in mice and, therefore, it is not commonly used as a criterion of virulence. Singer-Brooks [19] performed a comprehensive study on the effects of *Brucella* dose in mice. She observed that a larger proportion of mice succumbed after i.p. injections of massive doses of virulent smooth *Brucella* (> 4 × 10^8^ CFU/mouse), displaying obvious clinical signs. On the contrary, doses lower than 10^7^ CFU/mouse did not induce death or relevant clinical symptoms. Nevertheless, these non-lethal doses induced necrotic areas in the liver and spleen enlargement within the first 3 weeks pi. Perusal of the literature shows that these observations have been repeatedly confirmed.

The optimal dose of *Brucella* infection is defined as the lower number of bacteria that infects the spleen of all mice (between 20–23 grams) at consistent significant levels [97]. This optimal dose varies depending on the bacterial strain and route of infection as well as on the genetic background and physiological status of the mice. The optimal dose has been determined only for classical brucellae [25,35,75,81] as well as for some bacterial constructs [22,98]. When inoculated at low doses (< 10^3^ CFU/mouse), *Brucella* induces inconsistent infections in mice, generating wide standard deviations in CFU that complicate statistical interpretations. Doses lower than 10^3^ CFU/mouse do not produce gross anatomical changes, despite the fact that some mice show bacteria in several organs and tissues during the first weeks [19]. In contrast, very high *Brucella* inocula (> 10^7^ CFU/mouse) cause saturation of the spleen, to the point that the number of CFU per organ does not increase with respect to the optimal dose. Although these large doses induce a noticeable enlargement of the target organs (e.g. spleen and liver), distribution of *Brucella* in the reticuloendothelial system barely changes [29]; however, other organs may be also invaded [76]. If the mouse survives, the reduction of *Brucella* numbers follows its course albeit with a more protracted elimination time. *B. abortus* 2308 at i.p. doses between 5 × 10^8^ and 10^9^ CFU/mouse kill almost 50 % of the mice after 48 h, and 100 % before 1 week [48]. At these large doses, clinical symptoms such tachypnea, lethargy, piloerection, dehydration, and prostration were observed as early as 8 h pi. Since larger doses of killed *Brucella* are non-lethal, these symptoms relate to the massive organ invasion by live bacteria [48]. High numbers of attenuated *Brucella* (e.g. > 5 × 10^8^ CFU)*,* such as S19 or Rev1, seldom kill mice, although they may induce some clinical symptoms [22].

A relevant effect observed when inoculating large *Brucella* doses (e.g. > 5 × 10^7^ CFU/mouse) is that blood and cytokine profiles approach those induced by endotoxic bacteria like *Salmonella*[48]. This is an indication that *Brucella* neither induces an obvious inhibitory action on immune cells nor hampers the synthesis of proinflammatory cytokines at the onset of the infection, and that there is a threshold over which some molecules carrying pathogen-associated molecular patterns may be available to innate immunity receptors.

### The mouse

Mice are highly resistant to brucellosis because they are only killed by very large doses of virulent *Brucella*[19,48]. However, mice seem more sensitive to brucellosis than rats, hamsters and rabbits [13,99]. Mice infected with doses of virulent *Brucella* (e.g. *B. abortus* 2308) lower than 10^7^ CFU/mouse, hardly show any changes in behavior, or cachexia or wasting syndromes, all signs induced by endotoxic bacteria [19,48]. Nevertheless, and depending on the mouse strain, *Brucella* can cause long lasting infections that may extend throughout the lifespan, accompanied by characteristic pathological signs.

#### Resistant and susceptible mouse strains

All mouse breeds tested are susceptible to *Brucella*[49,100], suggesting the inexistence of specific resistance murine genes to brucellosis. However, some breeds seem more resistant than others (Figure 1b and Table 1). The best examples are the “susceptible” DBA2, C3H/He and BALB/c strains and the “relatively resistant” C57BL/10 and C57BL/6 derived strains [35,39,41,42,101]. The difference between these mouse strains is not related to blood clearance rates or to the number of bacteria reaching the spleen at the onset of the infection, which seem similar. Moreover, the resistance displayed by C57BL mice is not due to a stronger microbicidal activity of macrophages or to any other early innate immunity effector [102]. Rather, this resistance is manifested as different levels of splenic colonization one week after inoculation as well as dissimilar Th1 responses [103]. The spleen and liver CFU during the plateau phase (1 to 10 weeks pi) are commonly about ten-fold lower in the resistant C57BL strains (Figure 1B). In addition, the C57BL mice show less splenomegaly. The central difference between resistant and susceptible mouse strains seems to be the inability of the latter to maintain the production of IFN-γ after the acute phase, a phenomenon that extends throughout the chronic steady phase up to at least the 6^th^ week p.i. [65,103]. As expected, this phenomenon is redundant to other events, such as the recruitment and activation of immune cells [66,103]. A conspicuous difference between the BALB/c and C57BL/6 strains is the lymphocyte/granulocyte proportion in blood, which is close to 80/15 % in the former and to 90/9 % in the latter [20], a phenotype that may be related to *Brucella* clearance due to the regulatory action that neutrophils can display over macrophages [4].

*B. melitensis* replicates similarly in macrophages obtained from BALB/c and C57BL mice. Moreover, both strains of mice share the “sensitive” form (*Nramp1*^*s*^) of the *Nramp1*^*r*^ (natural resistance-associated macrophage protein) allele, which codes for a membrane phospho-glycoprotein implicated in the early activation of macrophages [43]. Unexpectedly, during the 1^st^ week after infection, the spleen and liver of “sensitive” BALB/c contain less *B. melitensis* CFU per organ than mice (e.g. C.CB) harboring the resistant *r1*^*r*^ allele form (Table 1). However, the spleen and liver weights in the *Nramp1*^*r*^ mice are larger than in the *Nramp1*^*s*^ mice, suggesting a more profound inflammation. It is noteworthy that expressing the results as CFU/gram of organ reduces the difference between these two strains. The absence of significant role of *Nramp1*^*s*^ allele in brucellosis is striking, since this gene is implicated in the resistance/susceptibility to other intracellular bacteria [104].

In spite of the quantitative differences, the *Brucella* replication profiles in the sensitive and resistant mouse strains follow a more or less parallel path [35,39]. This means that the slope of the replication curves is very similar in both strains (Figure 1B). Consequently, any *Brucella* strain displaying a more negatively pronounced slope must be considered attenuated, no matter whether the bacterium was tested in the resistant or in the sensitive mouse strain [22,37]. This parallelism between mouse strains is also maintained when testing attenuated brucellae [35,39]. Therefore, the differences in *Brucella* replication between the sensitive and resistant mouse strains should be interpreted in quantitative terms rather than in multiplication kinetics.

#### Mutant and knockout mice

Mice with defects or mutations influencing the innate or/and adaptive immune responses may show significant changes when infected with *Brucella* (Table 1). For instance, athymic nude mice do not clear *Brucella* after the acute phase [27,59], a time when cell mediated immune response has fully developed in immunocompetent mice [103]. During this period, nude mice develop granuloma in the liver and persistent infections of the biliary tract. In spite of this, the infection is not lethal (at least in 3 months), possibly because of immune compensatory phenomena existing in these mice [59]. In fact, nude mice seem to perform better in eliminating *Brucella* during the onset and early acute phases of infection. This suggests that the enhanced innate immune response displayed by these mutant mice [27,59] is able to partially control the infection, at least during this period. Moreover, nude mice are capable to develop a robust T-independent response against *Brucella* smooth lipopolysaccharide (LPS) [105]. Since antibodies against LPS are protective in murine brucellosis (Table 2), their generation in nude mice may well exert some protection at later times.

**Table 2:** **Effect on bacterial counts in mouse spleen (CFU) after passive transfer of antibodies, cells or cytokines at different phases of smooth virulent*Brucella*infection**

Treatment	Administration of treatment in relation to the time of infection	Main effect in infected mice	*Brucella* CFU in the spleen^a^		References
			Acute before 14 days	Chronic after 15 days	
Rabbit anti-*Brucella*	2 h, 16 h before or 2 h after	Immune serum from *Brucella* infected mice directed against a variety of different antigens	↓	↓	[106]
Murine anti-*Brucella*	2 h, 16 h before or 2 h after	Immune serum from *Brucella* infected mice directed against a variety of different antigens	↓	↓	[84,98,107]
Anti-LPS	16 h before	Immune murine sera against *Brucella* LPS	↓	↓	[84]
Anti-O:9	16 h before	Immune murine sera against *Yersinia enterocolitica* O:9. It protects mice but to a lesser extent than anti-*Brucella*	↓	↓	[84]
Anti-peptido-glycan	16 h before	Polyclonal immune sera against peptidoglycan protein complex, probably contaminated with LPS	↓	↓	[84]
Mabs anti-O- chain LPS	4 h before	Several antibody isotypes reacting against A, M and C epitopes of the *B. abortus* O chain of LPS	↓	↓	[35,108-110]
Mabs anti-Omps	24 h before	Against Omps of molecular weight 10, 16.5, 19, 25–27, 31–34, 36–38 and 89	↔	↔	[109]
Mab-anti-Omp16	24 h before	It induces lower protection than anti- O chain antibodies; IgG2a isotype	↓	ND	[109]
Mab-anti-Omp25	24 h before	It induces lower protection than anti-O chain antibodies; IgG2a isotype	↓	ND	[109]
Mab-anti-Omp2b	4 h before	Reacts against *B. abortus* Omp2b, which generally is not accessible in smooth bacteria	↔	ND	[35]
Mab-anti-Omp31	24 h before	It induces lower protection than anti-O chain LPS antibodies; IgG2a isotype	↓	ND	[109]
Spleen cells	Same day as infection	Protection was efficiently transferred to naive mice using spleen cells from mice infected 5 or 12 weeks earlier	ND	↓	[111]
Immune Tcells	2 h after infection	It gave similar protection than CD8+ or CD4+ cells passively transferred. Immune cells from six week infected mice. Before 4 week there is no protection.	↓	ND	[98,112]
Immune CD4+T cells	2 h after infection	It gave similar protection than CD8+ cells passively transferred. Immune cells from six week infected mice. Before 4 week there is no protection	↓	ND	[107]
Immune CD8+T cells	2 h after infection	It gave similar protection than CD4+ cells passively transferred. Immune cells from six week infected mice. Before 4 week there is no protection	↓	ND	[107]
Serum anti-*Brucella* and T cells	2 h after infection	Enhanced protection over administration of just T cells or Abs alone	↓	ND	[107]
Immune T cell+anti- INF-γ	Anti- INF-γ 1day before T cells with challenge	It gave similar protection than passively transferred T cells	↔	ND	[112]
Bovine Mø	1 day before infection	Transferred to NK1.1 cell-depleted Rag-1^−/−^ mice	↔	ND	[60]
Bovine Mø+γδT cells	1 day before infection	Transferred to NK1.1 cell-depleted Rag-1^−/−^ mice	↓	ND	[60]
Bovine Mø+CD4 T cells	1 day before infection	Transferred to NK1.1 cell-depleted Rag-1^−/−^ mice	↔	ND	[60]
INF-γ	1 day before and 2 and 4 day after	It Induces splenomegaly. Mice show enhanced peritoneal and splenic macrophage bactericidal activity	↓	ND	[113]
IL-12	With the infection and every 3 days after	The levels of INF-γ increase during the third week of infection	↔	↓	[114]
IL-1α	4 h before	CSF-1 increases in serum during the first 12 h. Colony forming cells increase in the spleen, mainly Mø and PMNs. Thirty days after treatment, the effect is terminated.	↓	↓	[28]
Transfer factor	At the sametime	No effect in immune enhancement or antibody response	ND	↔	[115]
Indomethacin	Daily s. c. for 7 days	Decrease of the cyclooxygenase activity by 80 to 90 % in spleen. Reduction of PGE2	↓	ND	[113]
Poly A:U	2 h before and 2, 4, and 6 days after	Activation of NK cell activity	↔	ND	[116]
Poly A:U	At the sametime	Polyadenylic acid-polyuridylic acid (poly A: U) is a non-toxic adjuvant that potentiates both humoral and cell-mediated immune responses	↓	↓	[27,117]
Cyclosporine	Daily for 4 weeks	It induces low inflammatory response in spleen and liver. No significant changes in spleen macrophage population	ND	↑	[107]
Corticosteroids	24 h before	It has a broad anti-inflammatory effects	↑	↑	[3]
Anti-Ia	24 h before	It depletes mostly B cells and some T cell subpopulation with “suppressor” activity	ND	↔	[111]
Anti-CD8+ T cells	5 days before and 3 per week	Depletion of CD8+ cells. DTH response was unaffected after treatment. Treatment abolished the IgG antibody response without affecting bacterial numbers.	ND	↑	[111]
Anti-CD8+ T cells	1 day before and every 4 days after	Depletion of CD8+ cells, significant increase of Møs in spleen. No significant effect in the number of CD4+, NK or γδ T cells	ND	↑	[118]
Anti-CD8+	1 day before and every 3 days after	Depletion of CD8+ lymphocytes involved in cell mediated cytotoxicity of infected cells	ND	↔	[119]
Anti-CD8+	2 days before and 1,4,7 10 days after	Depletion of CD8+ lymphocytes involved in cell mediated cytotoxicity of infected cells	ND	↑	[56]
Anti-CD4+	2 days before and 1,4,7 10 days after	Influences the Th1 profile mainly INF-γ. It induces basal levels of IL2 and IL4	ND	↓	[56]
Anti-CD4+		Reduces granulomatous inflammation, which seems to be mediated mainly by CD4+ T cells	ND	↔	[119]
Anti-CD25+ T cells	3 days before	Depletion of CD4+ regulatory T cells. Increase levels of INF-γ in spleen cells	ND	↓	[120]
Anti-NK1.1cells	24 h before	Depletion of NK cells and activity	↔	ND	[116]
Anti-asialo-GM1	24 h before and 3 day after	Depletion of NK cells and activity	↔	ND	[116]
Anti-PMN-RB6	24 h before and3, 6, 9 days after	It depletes neutrophils and a small population of Møs. It does not affect the course of brucellosis. In some cases CFU decrease in numbers after 9 days of treatment	↔/↓	ND	[48],unpublished results]
Anti-IL-10	1 day before and 4 days after	The levels of INF-γ increase during the first week of infection	↓	ND	[121]
Anti-IL-10	1 day before and 4 days after	Augments the production of INF-γ in spleen cells of both, sensitive and resistant mouse strains	↓	ND	[122]
Anti-IL-12	4 h before, or 2 days after, or 7 days after	Decrease in spleen weight and spleen inflammation in relation to infected non-treated mice. There is granuloma reduction and low levels of INF-γ	↑	↑	[123,124]
Anti-IL-4	24 h before and 4 days after	Removal of IL-4 It depresses the Th2 Ab response and indirectly may favor the Th1 response	↓	ND	[122]
Anti-INF-γ	1 day before infection	Reduces splenomegaly	↑	ND	[112]
Anti-INF-γ	1 day before and every 5 days after	No significant effect was observed even after administration with IL-12	ND	↔	[114]
Anti-INF-γ	1 day before and 4 days after	It removes secreted INF-γ and depressed Th1 response	↑	ND	[121]
Anti-INF-γ	24 h before and 4 days after	It removes secreted INF-γ and depressed Th1 response	↑	ND	[122]
Anti-TNF-α	1 day before and every 4 days after	No significant effect in the number of PMNs, CD4, CD8, NK, γδ T cells or Møs is observed	ND	↑	[118]
Anti-TNF-α	4 h before, or 2 days after, or 7 days after	Decrease in spleen weight and spleen inflammation with respect to the infected non-treated mice. INF-γ is detected at normal levels	↑	↔	[60,63,124]
Anti-TCRγδ	The same day and 3 days after	Removes Tγδ cells if innate immunity. Depletion has similar effect in IL/17Rα KO, INF-γ KO and GM-CSF KO mice	↑	ND	[60]

^a^ (↑) Significantly higher numbers of CFU in spleen than in controls; (↓) significantly lower numbers of CFU in spleen than in controls; (↔) No significant number of CFU in spleen in relation to the controls; ND, not done.

Virulent or attenuated *Brucella* extensively replicate in mice deficient in INF-γ production, a cytokine required to develop an adequate Th1 immune response (Table 1). When infected, the INF-γ deficient mice show significant clinical signs, such as cachexia and a severe splenomegaly, with macrophages accounting for more than 75 % of the spleen cells; these mice eventually die [118]. Similarly, *Brucella* replicates extensively in knockout mice defective in IFN-γ regulatory factor (IRF)-1 or in mice displaying mutations in the IFN consensus sequence binding protein (ICSBP), which are transcriptional elements regulated by INF-γ [53]. Analogous to what has been observed in the INF-γ deficient mice, *Brucella* (e.g. > 5 × 10^5^ CFU/mouse) are lethal for IRF-1 mutant mice. In these animals, not the spleen but the liver is the main target organ [53]. Furthermore, whereas the liver shows significant hepatitis and granuloma formation, the spleen yields CFU numbers similar to those obtained in the parental immunocompetent mice. *Brucella* also replicates better at later times in mice defective in regulatory cytokine IL-12 (Table 1), involved in maturation of T cells and necessary for the development of Th1 immune responses [53].

Mutations in genes coding for IL-1β, IL-18, TLR5, TLR2, NOD1, NOD2, GM-CSF, IL/17r, Rip2, TRIF or type-1 INFr, all key factors of innate immune response, have little or no effect on *Brucella* spleen replication (Table 1). However, mutations disturbing TLR9, Myd88, IRAK-4, Tγδ cells, or in the generation of TNF-α, influence the clearance and favor *Brucella* replication (Table 1). There is some controversy on the role of TLR4 in murine brucellosis. While two studies [44,47] detected a slightly higher susceptibility in TLR4 knockout mice, others have found that the absence of TLR4 does not influence *Brucella* replication (Table 1). *Brucella* LPS signals through TLR4 but very inefficiently [50,125] and the reported susceptibility associated with TLR4 is not as large as that observed for other Gram-negatives [48]. Mutations in the iNOS and *gp91*^*phox*^*,* which affect several innate immunity pathways, are not lethal and favor the replication of *Brucella*, mainly at later times (Table 1). Hybrids harboring double mutations in some of these genes (e.g. iNOS/ICSBP) display profound deficiencies that favor *Brucella* replication (Table 1).

Defects in the adaptive immune response generate divergent phenotypes (Table 1). For instance, mutations such as *rag1,* which hampers the maturation of some populations of B and T cells, do not have significant influence in *Brucella* replication. In the case of the *igh6* mutation that impedes the development of B lymphocytes, there are contradictory reports: while some authors detect a decrease of *Brucella* CFU in the spleens of *igh6* mutant mice after the 1^st^ week [55], others do not notice significant changes at early times, and barely some decrease at later times [54]. On the contrary, disruption of *β2m* or perforin (*pfp*) genes (that impact on the development and function of cytotoxic CD8+ T cells) seems to favor *Brucella* replication. However, there are reports indicating that the *β2m* mutation either does not have any influence [55] or supports the elimination of *Brucella* later in the infection [54]. Nonetheless, *rag1* and *igh6* knockouts have problems in eliminating extracellular non-virulent *Brucella* VirB mutants [126], suggesting that these genes may play some role [54].

Knockout mice showing other immune defects do not yield clear-cut results (Table 1). For example, mice with defects in IRF-2 (a transcriptional factor regulated by INF-γ), *Cd4* (with a defect in the CD4+ T subset), or Aβ (MHC-II deficient) seem to eliminate *Brucella* more efficiently than the parental strains. However, some authors do not report changes in spleen CFU in Aβ deficient mice [55]. IRF-2 knockout mice also have a conspicuous defect in NK cells, but these cells do not play a relevant role against *Brucella* infection [53]. The absence of CD4+/CD25+ regulatory T cells (involved in the down regulation of T cells) in *Cd4* and Aβ mutant mice may balance the response towards Th1, thus favoring the elimination of *Brucella*[54].

From experiments in mutant and knockout mice a few general conclusions may be drawn. For instance, several factors of the innate immune system that in other bacterial infections play an essential role, seem to be of minor importance (e.g. iNOS, type-1 INFr, and *gp91*^*phox*^) or irrelevant (e.g. IL-1β, IL-18, TLR4, TLR5, TLR2, NOD1, NOD2, GM-CSF, IL/17r, Rip2, TRIF, NK or *Nramp1*^*s*^) in murine brucellosis. It is also clear that Th1 response via INF-γ is crucial for controlling *Brucella* replication and that any event that negatively influence the generation of this cytokine (e.g. *ifng*, IRF-1, IL-12KO) severely compromise the overall adaptive immune response against brucellosis. Finally, the absence of some cell populations of the immune system (e.g. *igh6,* IRF-2, *Cd4* and Aβ) seems to favor the elimination of *Brucella*, a fact that suggests that some regulatory phenomena are induced during infection. All these events are in agreement with the evolutionary stealthy strategy that *Brucella* has followed to hide from and modulate the immune system [4,48,125].

#### Age and sex

In the only study published on the influence of age [30], it was reported that a *B. abortus* 2308 dose of 4 × 10^8^ CFU/mouse was similarly non-lethal for 2 and 18 month old DBA/2 mice, and that a ten-fold higher dose killed all animals in both age groups. Although such clear-cut differences between these two very high doses of a virulent strain are striking (see Infective Dose, above), these results may indicate that age does not significantly influences the susceptibility to lethality by *B. abortus*. However, whereas the bacterial counts in the spleen remained relatively high and stable for at least 8 weeks in the young adult mice, the numbers decreased in the spleen of older mice after the 5^th^ week. In the same work, it was reported that the anti-*Brucella* immune responses in older mice were less-Th1 specific and showed higher levels of IL-17, and the authors suggested alternative pathways for combating brucellosis in aged mice.

With the exception of the *Brucella* "resistance" character of C57BL mice, which seem to be partially dominant with polygenic control in females [41,42], no comparative studies have been performed between sexes. The placenta becomes infected in pregnant female mice and the testes constitute a site of *Brucella* colonization in the case of males [74,76,127,128], two facts that are reminiscent of the events in domestic ungulates and humans with brucellosis [129,130].

#### Transmission

*Brucella* horizontal and vertical transmissions are common in natural hosts [131] but rare in mice and humans [132,133]. Although the mammary glands of nursing dams are colonized with *Brucella,* less than one percent of the mouse pups become infected [132,133]. Similarly, *Brucella* colonizes salivary glands, kidneys and testes, but venereal transmission or contagion rarely occurs [25,76]. This is striking since rats (closely related to mice) shed the bacteria in the urine and they are prone to transmit *Brucella* by the venereal route [134].

Transmission of virulent *B. abortus* 544 from the mother to the fetus was demonstrated in mice [25,133], with profuse infection of placentas [127]. Although mice seem to be quite resistant to abortion, this event can be induced at specific time periods. Attenuated *B. abortus* S19 is also transmitted to the fetus, but it seldom induces abortion [135]. These two events are somewhat reminiscent of what happens in the natural host [136].

## Physiopathology

The inoculation of virulent brucellae induces clinical and physiopathological responses in mice that differ from those caused by attenuated or non-virulent *Brucella* strains [53]. These responses are less severe in *Brucella* vaccinated or immunostimulated mice and more conspicuous in pregnant or immunodeficient mice (Table 1). In the following paragraphs, the main events taking place during different infection phases are described (Figure 1A).

### Onset of the infection

Optimal doses (e.g. 10^4^ to 5 × 10^6^ CFU/mouse) of virulent brucellae by the i.p. or i.v. routes barely induce physiopathological symptoms at early stages of infection. The absence of obvious clinical signs correlates with: i) normal blood cell and platelet counts; ii) the lack of a recruitment of proinflammatory cells at the site of infection; iii) the presence of minimal levels of serum IL-1β, TNF-α, IL-10 and IL-6; iv) very low amounts of MCP-1 and RANTES chemokines [48,137]; and, v) the absence of synthesis and degradation of fibrinogen and coagulopathies [48]. IL-10 is not detected in serum and its corresponding transcript only appears after 3 days of infection [126]. Although IL-10 may be extracted from murine spleen cells after 1 day of infection [138], the levels of this cytokine are far lower than those induced by other bacteria [139,140]. This suggests that the regulatory role of IL-10 is minor or irrelevant at early times of *Brucella* infection. At these early times, INF-γ and IL-12, are barely detected in serum or cell extracts and these cytokines become evident only during the next infection phase [63,135,137,138,141]. However, the low levels of INF-γ and IL-12 are not unique to early *Brucella* infections, since they are also observed at the onset of murine salmonellosis [142]. Although transcripts of CXCL1 and MIP-2 chemokines, and IL-6 can be detected in spleen cells one day after infection [65], their levels are significantly lower than those induced by other bacteria. Anti-*Brucella* antibodies and IL-4 are not detected in serum or in spleen cells at the onset of the infection [138,143]. In summary, the proinflammatory response to virulent brucellae is very low and it may have some significance only when compared with that induced by non-virulent strains such as VirB or BvrS [37,126].

A few hours after infection, *Brucella* is detected inside phagocytic cells in the blood, spleen, liver and bone marrow of mice [14,15,76]. In the liver, bacteria are detected in sinusoids and within Kupffer´s cells as early as 3 h after i.p. inoculation. During the first 6 h, neutrophils gather around macrophage Kupffer´s cells; thereafter, the number of bacteria decreases and seems to disappear due to engulfment by liver phagocytic resident cells, which become engorged with intracellular brucellae [14,15]. During the early phase of infection, spleen macrophage, neutrophil and colony forming cell numbers are not significantly different from those of non-infected mice [28,43].

At the onset of infection, a normal distribution of spleen cells is observed with some minor congestion and presence of T cells, preferentially located in the periarteriolar lymphatic sheaths and within the red pulp. Concomitantly, B lymphocytes are mostly present in the corresponding B-cell zones, marginal zones and red pulp, while granulocytes and macrophages are scattered in the red pulp and marginal zones. Activated phagocytes expressing iNOS are not observed at these early times of infection [65]. Treatment of mice with the immune enhancer poly A:U 1 h before infection does not affect the number of CFU/spleen during the first 24 h. Nevertheless, it promotes the elimination of the bacteria after 48 h. This is an indication that an early activation of the innate immune system is detrimental for *Brucella* multiplication and that, when professional phagocytes are properly activated in vitro or in vivo*,* they are capable of eliminating the invading *Brucella*[48,117].

### Acute phase

This phase (from the 3^rd^ day to the 2^nd^-3^rd^ week) is marked by the rapid increase of bacterial numbers in the target organs, a significant inflammation of the spleen and lymph nodes and the appearance of the first pathological lesions in the liver. In addition, there is a development of type IV delayed type hypersensitivity (DTH), corresponding with the beginning of the Th1 response [65,144]. *Brucella* can be readily isolated from blood and many organs. However, as time passes by, it becomes more difficult to find the bacteria in blood. Organ cell infiltration becomes significant at the end of this phase, with augmented frequencies of phagocytic cells [14,15,43,65]. During the acute phase the non-gravid uterus, lungs, heart, kidneys, brain and gastrointestinal tract do not show conspicuous pathological signs [145].

**Figure 3:**
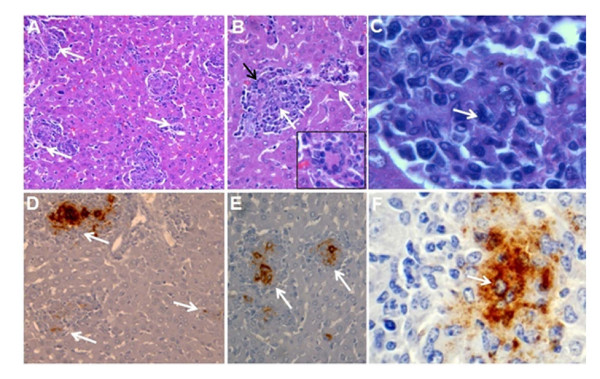
**Liver pathology and intracellular detection of *Brucella *antigens in macrophages of BALB/c mouse after 10 days of infection with virulent* B. abortus *2308.** (**A**) Liver granulomas (pointed by white arrows). (**B**) Large and smaller liver granulomas (white arrows) with giant cells (black arrow and insert). (**C**) Mononuclear infiltrate formed mainly by macrophages and histiocytes (white arrow). (D-E) immunoperoxidase detection of *Brucella* LPS antigen in matching histological sections of the corresponding upper A, B and C panels. Hematoxylin-eosin stain (A-C) and hematoxylin counterstain (D-F).

The liver is the first organ to show significant histopathological changes. Mild perivascular mononuclear infiltrates are observed after the 3^rd^ or 4^th^ day of infection with virulent *Brucella*, because of the localization of bacteria inside Kupffer´s cells. Thereafter, granulomas become conspicuous, reaching their maximum intensity after the 1^st^ week of infection (Figure 3). Granulomas are composed by clusters of macrophages and dendritic cells [146], generally known as epithelioid cells and histiocytes, several of which demonstrate ingested material as well as *Brucella* antigens (Figure 3). The presence of plasma cells and lymphocytes becomes evident, but very little or negligible granulocyte infiltration is present in the liver in this phase. At this stage, liver pathologies induced by the virulent *B. abortus* 2308 and the attenuated S19 strain are not significantly different. Mice infected with non-virulent *Brucella* BvrS/BvrR or VirB mutants do not generate significant pathological responses in the liver or the spleen at any stage of the infection [126], and Grilló, Blasco and Moreno, unpublished results]. However, some immunodeficient mice, like the IRF-1 mutants, develop more and larger liver granulomas during the acute phase when infected with virulent or attenuated *B. abortus*[53].

**Figure 4:**
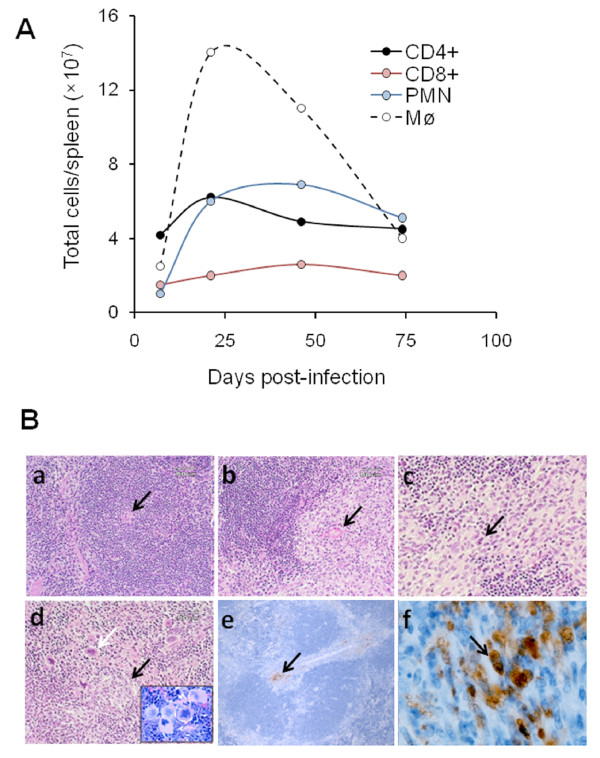
**Spleen cell population profiles and histopathology after infection of BALB/c mice with*B. abortus *2308.** (**A**) Spleen cell populations. The total number of CD4 T cells, CD8 T cells, neutrophils (PMN) and macrophages (Mø) per spleen was determined by multiplying the percentage of positive cells obtained by differential microscopy observation after cytospin centrifugation and fluorescent flow cytometry analysis by the total leukocyte count. Standard deviation at all points is lower than 10 % of the respective value (adapted from [57]). (**B**) Spleen histopathology and detection of *Brucella* antigens in the spleen. (**a**) Normal spleen (arrow points to the central artery). (b-f) Histological sections of spleen nodules during the acute phase of infection with virulent *B. abortus* 2308 (**b**) Spleen nodule with a clearer area infiltrated by macrophages (arrow points to the central artery). (**c**) Macrophage and histiocytes (arrow) infiltrating the spleen nodule. (**d**) Active extramedullary hematopoiesis (white arrow and insert) with granulomas (black arrow) in some areas of the spleen. (**e**) Immunoperoxidase detection of *Brucella* LPS antigen around the central artery of a spleen nodule. (**f**) Immunoperoxidase detection of *Brucella* LPS antigen within macrophages (arrow).

During the 1^st^ week of infection with virulent *Brucella*, the spleen sizes increases (Figure 2A), showing a mild lymphoid depletion in the splenic nodules, moderate macrophage infiltration, few neutrophils and a mild extramedullary hematopoiesis in the red pulp [64]. In spite of this, the overall number of macrophages and neutrophils remains practically unaltered [43]. As expected, higher doses of smooth brucellae (e. g. > 10^7^ CFU/mouse) induce larger inflammation [19,145]. During the 1^st^ week of the acute phase, the numbers of macrophages, neutrophils, CD4+, and CD8+ T cells remain grossly within the limits of uninfected spleens (Figure 4) [57,65,118]. After 10 days of infection, the spleen size increases as the number of CFU augment and the degree of lymphocyte depletion, macrophage infiltration and extramedullary hematopoiesis displaying mitotic cells is prominent. Some macrophages may have intracellular *Brucella* antigens.

At the end of the acute phase, the number of macrophages and neutrophils in the spleen increases slightly (Figure 4A) [43]. While the B-cell areas remain populated and the CD4+ and CD8+ T lymphocytes have decreased in the splenic nodules, the T-cell zones have been displaced by macrophages [112]. The overall number of B cells and CD4+ and CD8+ T cells in the spleen is slightly higher than in spleens of uninfected mice [57,64,112,118]. Nevertheless, the confined depletion of lymphocytes seems to be relative to the spleen swelling, rather than to a true decrease in cell content [147].

During the acute phase there are just a few but significant differences between the spleen cell profiles induced by the virulent *B. abortus* 2308 in comparison to that generated by the attenuated vaccine S19. One week after inoculation, S19 produces a relatively more severe local lymphoid depletion than strain 2308 [64]. This corresponds to a slightly larger spleen size in S19 infected mice (Figure 2A), which also displays more intense neutrophil infiltration [64]. However, the most significant differences in the pathological signs induced by virulent and attenuated vaccine strains are evidenced at the end of the acute phase and in the next phase. Non-virulent brucellae (e.g. BvrR/BvrS) fail to induce significant spleen changes and hardly any signs of inflammation.

**Figure 5:**
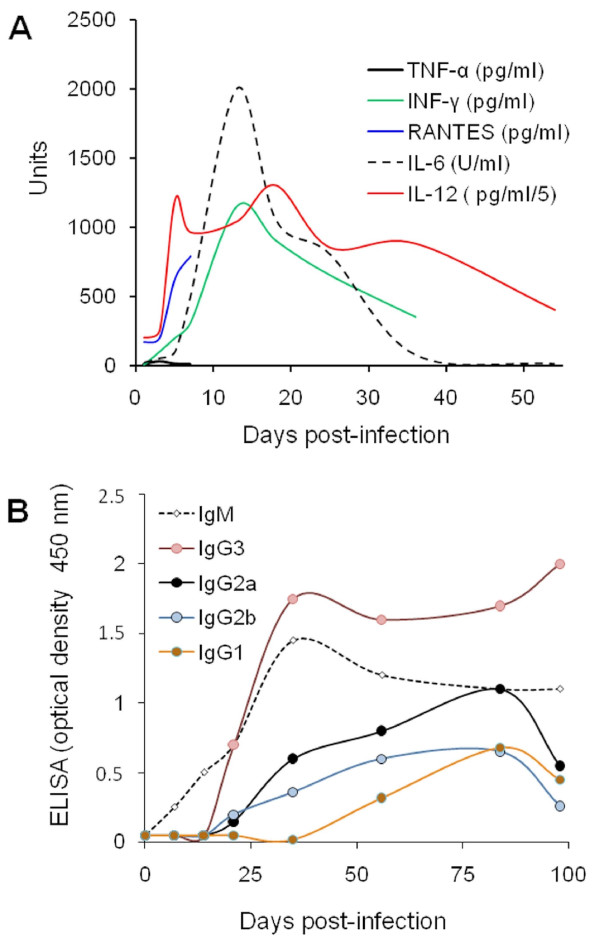
**Cytokine and antibody serum profiles of *B. abortus *2308 infected mice.** (**A**) Serum cytokine levels in BALB/c mice after infection with virulent *B. abortus* (INF-γ, TNF-α, RANTES) or attenuated vaccine S19 (IL-6, IL-12) stains (adapted from [135,137,148,149]). (**B**) Antibody response of virulent *B. abortus* 2308 infected CD-1 mice (adapted from [138]). Notice that in “A” the absolute units are different for each cytokine, according to the indication (e.g. while TNF-α, INF-γ, RANTES are measured in pg/mL, IL-12 are in pg/mL/5 and IL-6, in Units/mL). For clarity, the SD were not included.

After the 1^st^ week of the acute phase, significant amounts of INF-γ, IL-12, IL-6 and RANTES are present in sera of susceptible mice (Figure 5) [114,135,137,141,148,149]. After the 2^nd^ week, these cytokines steadily decrease approaching basal levels by the 6^th^ week, already in the next infection phase (Figure 5A). The endogenous IL-12 extracted from spleen cells of infected mice seems to parallel the kinetics in sera, although at lower levels [138,148]. Similarly, endogenous INF-γ (and its transcript) attains maximum levels during the first 2 weeks of the acute phase and is still detected (albeit at significant lower levels) after 8 weeks, in contrast to endogenous IL-12 [65,138]. The difference between serum and endogenous INF-γ suggests that it may still remain as a reservoir pool inside cells of susceptible mice, but not released into circulation at later times. In contrast to what happens in the susceptible BALB/c mice, the resistant C57BL/10 strain does not display INF-γ in serum during the acute phase [114]. Moreover, the INF-γ kinetics profile depends on the *Brucella* virulence. For example, INF-γ levels decrease faster after inoculation with attenuated S19 than after infection with virulent *B. abortus*[65,141].

Cultured spleen cells from infected mice are able to generate cytokines after ex vivo challenge with *Brucella* antigens. In this ex vivo protocol the kinetics of IFN-γ parallels those of GM-CSF and IL-10 production, displaying an early rise by the 3^rd^ or 4^th^ day after infection, reaching peak levels between days six and ten, and then declining sharply [65,150,151]. Regardless of whether the assays are performed in sensitive or resistant mouse strains, both IL-12 and IFN-γ are produced ex vivo during secondary stimulation of cultured spleen and CD4+ T cells with *Brucella* antigens during the 1^st^ week of infection [92,114,122]. However, by the 3^rd^ week of infection, at the beginning of the next phase, there is a decrease in IL-12 receptor-2 expression in spleen cells of the susceptible BALB/c mice, corresponding to their inability to produce IFN-γ at later times [114,122]. In this mouse strain the IFN-γ levels remain low until the end of the next phase, close to week 10 pi. Then, the spleen cells can be specifically restimulated with *Brucella* antigens to generate this cytokine [65].

During the acute phase, the production of IL-18 (which works synergistically with IL-12 to induce the generation of INF-γ) is depressed in spleen cells of *B. abortus* infected mice [144]. Therefore, once the infection has been established, the limited secretion of IL-18 does not affect the endogenous production of IFN-γ. Simultaneously, small amounts of endogenous IL-10 (and its transcript) reach their maximum during the 1^st^ week of the acute phase, disappearing from the splenocytes during the 2^nd^ week pi [138]. It may be that the endogenous synthesis of IL-10 could, after all, influence the production of IFN-γ and the premature development of the Th1 response.

**Figure 6:**
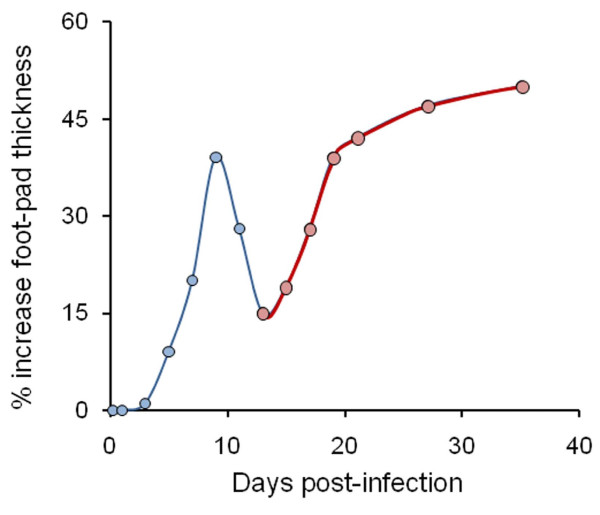
**DTH in *B. abortus* S19 infected mice after footpad injection of *Brucella *protein extracts.** Note the biphasic response between the acute and chronic steady phases. The blue line in the graphic is compatible with type IV hypersensitivity, while the red line is compatible with a mix reaction of type IV and type III hypersensitivity (adapted from [153], with permission).

**Figure 7:**
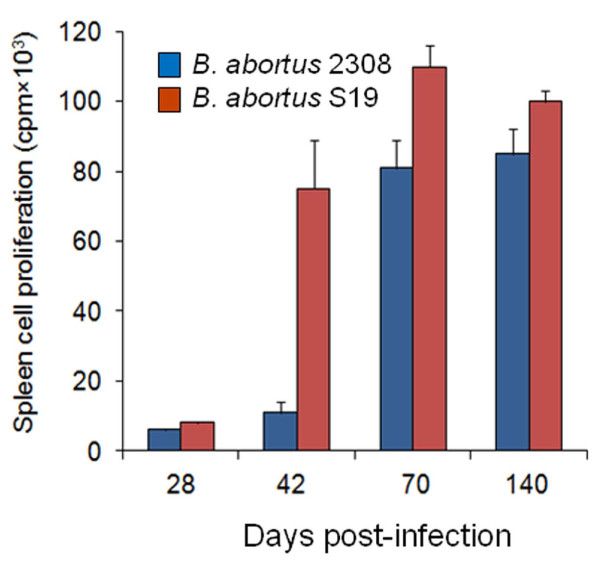
**Spleen cell proliferation (**^**3**^ **H-thymidine incorporation) in response to killed* B. abortus* 2308 in BALB/c mice infected with* B. abortus* virulent 2308 or vaccine-attenuated S19 strains, during 20 weeks [adapted from [**[152]**] with permission].** Notice the delay in response of spleen cells from 2308 infected mice in relation to those infected with the attenuated strain S19.

Although anti-*Brucella* antibody producing cells are present in the spleen early after infection, relevant levels of anti-*Brucella* immunoglobulins are detected only after the 2^nd^ week pi (Figure 5B), with relatively higher levels of IgG3 [30,65,67,138]. DTH to *Brucella* antigens becomes evident during the acute phase [153], reaching its maximum at 9 days pi (Figure 6). The second event (Figure 6, red line) observed after the 2^nd^ week may correspond to a mixture of type III and IV hypersensitivity reactions. However, macrophages display their maximum unspecific killing activity at 18 days pi, at the end of the acute phase and persist for 4 weeks, albeit, at lower levels. This phenomenon is known as the Mackaness effect [153], described as “an immune response specifically induced but non-specifically expressed”. In addition, cultured spleen cells from *Brucella* infected mice do not proliferate in response to challenge with killed *Brucella* or soluble antigens (Figure 7) [152]. This suggests the presence of regulatory phenomena at this stage [4]. Only negligible amounts of TNF-α (Figure 5A), IL-4 and MCP-1 are present in the sera of *Brucella* infected mice in the acute phase and thereafter [38,63,135,137,138]. IL-2 and IL-4 are barely detected in spleen cells from infected mice stimulated with *Brucella* antigens during the acute phase [92,122,151]. The quality of *Brucella* antigens profoundly influences the outcome of the immune response. Cultured spleen cells from mice infected with live *Brucella* display a Th1 response marked by INF-γ and IL-12 production. However, spleen cells from mice immunized with soluble *Brucella* antigens generate preferentially a Th2-like response, with IL-4 and IL-2 production by CD4+ T cells [151]. In addition, there is a higher frequency of precursor IFN-γ-producing CD4+ T cells and a lower frequency of precursor IL-4-producing CD4+ T cells in *B. abortus* infected mice than in mice immunized with *Brucella* soluble antigens [151].

#### Acute phase in pregnant mice

Pregnant mice offer a special environment for *Brucella* replication [145]. Murine brucellosis during pregnancy has been explored mainly throughout the acute phase, because the mouse gestation time has an average of 19 days. *B. abortus* (i.p. 10^4^ CFU/mouse) induces higher “abortion” rate (death pups on day 18.5, before natural delivery) when administrated on day 4.5 of pregnancy than when injected at later times of the gestation [135,137,154]. The degree of colonization and placental damage depends on the doses and the pregnancy period. After the 7^th^ day of pregnancy, doses lower than 2 × 10^5^ CFU/mouse seldom induce miscarriages or fetal deaths, independently of the infection route. This is an indication of the mouse resistance against *Brucella*-induced abortion [74,127,132,133]. However, in close parallelism to the events in natural hosts, placentas are more intensively colonized when mice are challenged during mid pregnancy (days 7–11) than when inoculated during early (e.g. day 3) or late (e.g. day 15) pregnancy [135,137,154]. Placental colonization and abortion are not always linked. In some experiments, virulent *B. abortus* 2308 colonizes the placenta without inducing abortions, although it may cause fetal deaths [145]. When mice are infected at day 9 of gestation, the invaded placentas have lost weight, look edematous and frequently harbor pale and shrunken autolyzed fetuses 9 days later (day 18 of pregnancy) [145]. Strikingly, mice born alive from infected dams do not demonstrate gross macroscopic or microscopic alterations [145], and no differences in bacterial loads between the live and aborted fetus are detected [135]. All these observations suggest the existence of refractory “placental windows” to *Brucella* infection. Attenuated (e.g. S19) and non-virulent *Brucella* (e.g. VirB mutants) seldom induce abortions, although S19 may cause restricted placental infections [38,137].

**Figure 8:**
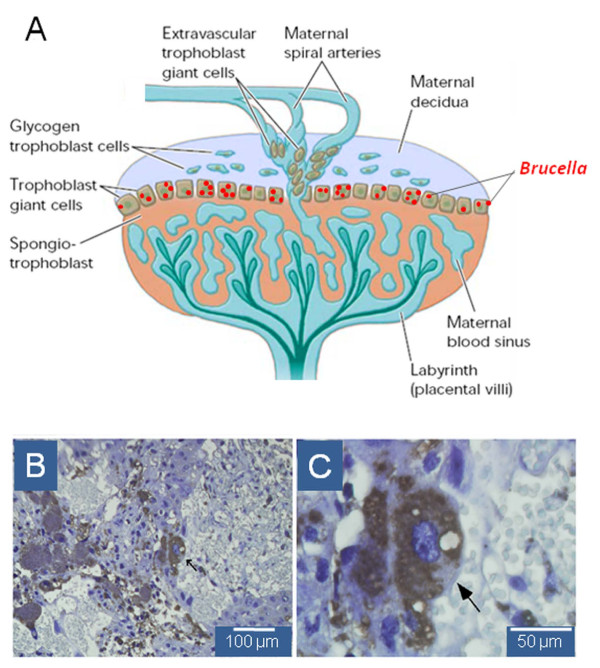
***Brucella *invasion of mouse placenta.** (**A**) Model of a mouse placenta with trophoblast giant cells (in ocher) infected with *Brucella* (in red). (**B**) Immunochemical detection of intracellular *Brucella* inside giant trophoblasts (arrows) of ICR mice infected with virulent *B. abortus* 544, at 15 days of gestation; Meyer's hematoxylin stain. (C) Indicate the magnified image from panel (B) (adapted from [135], with permission).

*Brucella* replicates within giant trophoblasts located in the *deciduas basalis*, 3 days after infection of mice in the 12^th^ day of pregnancy (Figure 8) [135]. Two days later, most bacteria are already found within giant trophoblasts, and necrotic foci become evident within the spongiotrophoblastic zone. Inflammatory cells are mainly found along the regressing layer of the endometrium overlying the implanted chorionic vesicle, or free within the newly formed uterine lumen [135]. The occurrence of neutrophils is likely the result of tissue destruction in necrotic areas. Indeed, when bacteria are solely located within giant trophoblasts with no cell destruction, neutrophilic inflammatory response is not observed. This confirms the absence of granulocyte recruitment by *Brucella* organisms at the site of infection [48]. A multifocal necrosis of the spongiotrophoblastic zone of the placenta coalescing in several zones is produced, 7 to 9 days after infection (corresponding to 16 to 18 days of gestation) [135]. In this region, which extends from Reichert’s membrane at the periphery of the disk to the interior, extracellular bacterial colonies are present together with a few giant trophoblasts remaining infected. Throughout the necrotic regions, there is massive bacterial colonization and phagocytosis of *Brucella*. In some placentas, thrombosis of the uterine vessels in the junctional zone resulted in infarction of the labyrinth zone. All these lesions resemble those observed in the placentas of *Brucella* infected natural hosts [89].

Abortion in mice seems linked to INF-γ increase, RANTES production and to low expression of heme oxygenase-1 in the giant trophoblasts [135,137,154]. Neutralization of INF-γ and RANTES inhibits abortion in mice inoculated with *Brucella* at day 4.5 of gestation. Moreover, down-regulation of heme oxygenase-1 expression in giant trophoblasts is enhanced by IFN-γ treatment. TNF-α or MCP-1 are not involved in *Brucella* induced abortion in mice. Non-virulent *Brucella* VirB mutants do not lower the amounts of heme oxygenase-1 in murine giant trophoblasts and barely induce production of INF-γ, RANTES.

### Chronic steady phase

The chronic steady phase (from the 3^rd^ to the 8^th^–11^th^ week) is noticeable by high levels of infection, describing a plateau with a maximum and sustained number of CFU in the target organs (Figure 1A). During this phase, bacteremic episodes are transient and the chances to isolate *Brucella* form blood are scarce. The liver granulomas increase in size, mainly because of the merging of disperse smaller granulomas developed in the acute phase [59]. At this stage, macrophages contain *Brucella* antigens, indicating bacterial destruction within phagocytes [59]. Some macrophages fuse and become polykaryons and multinucleated giant cells within the well demarcated liver granulomas. Commonly, these giant cells are located in the granuloma centers and contain from 5 to 20 nuclei (known as Langhans cells). The bone marrow of infected mice also shows granulomas but the lungs, heart, kidneys or gastrointestinal tract do not show significant pathological lesions [88,145]. The appearance of granulomas and giant cells in the liver seems to correspond to an innate phenomenon not mediated by T cells. Indeed, granulomas and giant cells equally occur in normal and athymic nude mice [59]. The number and size of liver granulomas are larger in mice infected with virulent *B. abortus* than with attenuated S19. Moreover, mice infected with S19 seldom display giant cells. Unless deeply immunosuppressed, mice infected with non-virulent *Brucella* do not enter into this phase; therefore, granulomas are rarely present.

**Figure 9:**
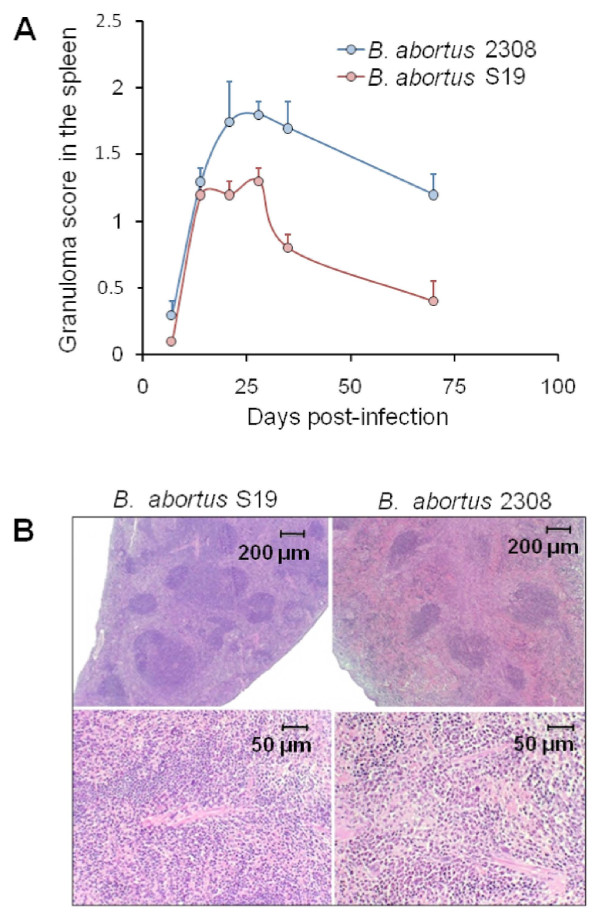
**Spleen inflammation after infection of BALB/c mice with attenuated* B. abortus* S19 vaccine strain or virulent* B. abortus* 2308 (A) Generation of granulomas in the spleen (adapted from [**[64]**], with permission).** (**B**) Histological sections stained with hematoxylin-eosine of spleens of CD-1 mice infected with *B. abortus* S19 after 6 weeks and with *B. abortus* 2308 after 8 weeks. As shown in Figure 2a, the spleen of S19 inoculated mice after 6 weeks of infection is considerably smaller than those infected with 2308. The proportion of the white pulp has been reestablished and the number of macrophages and neutrophils in the periphery of the central arteries of the nodules has considerably diminished in the spleens of S19 infected mice in relation to those infected with virulent 2308 strain, which is hyperemic and infiltrated with inflammatory cells.

After the 2^nd^ week of infection and parallel to spleen swelling (Figure 2B), granulomas and giant cells increase in this organ, until the middle of the chronic steady phase (5^th^ to 6^th^ week) (Figure 4B). Thereafter, there is a slow decrease in the size and number of granulomas. This becomes evident at the end of the chronic steady phase (Figure 9) [64]. During the 3^rd^ week pi, the apparent depletion of lymphocytes in the white pulp reaches its maximum. Then, after the 4^th^ week, there is a gradual increase in lymphoid hyperplasia and extramedullary hematopoiesis with several mitotic figures and multifocal accumulation of macrophages that surround and sometimes cover the periarteriolar lymphoid sheaths (Figure 4B). In spite of the apparent lymphocyte local depletion, the total number of CD4+ and CD8+ cells increases moderately (two and one fold, respectively) about the 3^rd^ week of infection (Figure 4A) [57,118]. Along with this, infiltrating macrophages and neutrophils increase to relatively large proportions (seven and eight fold, respectively) (Figure 4). This increase is proportional to the swelling and infiltration of blood and phagocytic cells in the spleen. However, there is some controversy regarding the suggested lymphocyte depletion in the spleen of infected mice [65]. Morphometric and histopathologic analysis of spleens of *B. abortus* infected mice do not reveal significant decrease in lymphocytes in the white pulp [147]. This indicates that the apparent depletion of lymphocytes is a local phenomenon related to the inflammation and influx of blood, rather than an absolute decrease of these cells.

At the end of the steady chronic phase, the splenomegaly has already decreased (Figure 1A) and splenocytes come close to normal numbers and distribution [57,64]. Parallel to the reduction of macrophage infiltration and B cells repopulation of the germinal centers, the CD4+ and CD8+ T lymphocytes recolonize the periarteriolar lymphatic regions [65]. By this time, colocalizing *Brucella* antigen and iNOS-positive activated macrophages are observed in periarteriolar lymphatic sheaths [65]. At these later times, *Brucella* antigens confined in macrophages are still present in the spleen [152]. However, there is no significant change in MHC-I or MHC-II expression on the surface of spleen macrophages [118].

After the 4^th^ week, the differences between spleens of mice infected with virulent or attenuated *B. abortus* S19 become evident [2]. The number of spleen granulomas in mice infected with virulent *B. abortus* is significantly higher (Figure 9), displaying extensive hyperplasia of the periarteriolar tissue. In addition, extramedullary hematopoiesis, neutrophil and macrophage infiltration are significantly higher in mice infected with virulent *Brucella*. Following the decline of S19, a decrease in splenic granulomas, neutrophil infiltration and extramedullary hematopoiesis is observed. However, the number of macrophages remains high and lymphoid hyperplasia is evident (Figure 9). The emergence of large germinal centers in the spleen with reduction of macrophage accumulations does not occur before 6 to 10 weeks pi with S19, and after 10 weeks pi with the virulent strains. The presence of antigen in macrophages persists longer in mice infected with virulent *B. abortus* than in those inoculated with S19 [152].

In addition, spleen cells of mice infected with S19 readily proliferate at 6 weeks pi while the splenocytes of mice inoculated with virulent *B. abortus* multiply later (Figure 7) [17,152]. This phenomenon (linked to the virulence of *Brucella* strains and to the severity of the infection) is consistent with the observed pathological changes of the spleen and suggests some inhibitory action related to the presence of regulatory CD4 + CD25+ T cells [4,120]. This is also in agreement with the tendency of spleen lymphocytes from mice repeatedly immunized with *Brucella* antigens to produce less INF-γ when stimulated in culture with the cognate antigen [155]. It is worth noting that cultures of spleen cells proliferate in response to *Brucella* antigens 2 weeks earlier when mice are infected with the attenuated rough *B. abortus* RB51 than when infected with S19 [152]. This event seems to be related to the lesser spleen inflammation and faster elimination of the rough strain in relation to smooth bacteria. It seems, therefore, that in murine brucellosis the immune regulatory events are linked to the overall bacterial virulence, rather than to specific bacterial mutations.

infected BALB/c mice produce substantial amounts of serum IFN-γ during the acute phase but much less during the chronic steady phase (Figure 5A). On the contrary, in the resistant C57BL/10 serum IFN-γ is just detected in significant amounts at latter phases (50–75 days), corresponding to bacterial clearance in this strain of mice [114]. The lack of measurable IFN-γ in the serum of infected mice does not necessarily mean that cells are not producing this cytokine in response to *Brucella* infection (in order to detect IFN-γ in serum, relatively large amounts of this cytokine need to be produced) [114]. Nevertheless, in clear contrast to the C57BL resistant mouse strain, the levels of IFN-γ produced ex vivo by BALB/c spleen cells are about two fold higher during the 1^st^ week of the acute phase. Then, a hiatus in IFN-γ production is observed in the sensitive but not in the resistant mice at the beginning of the chronic steady phase [118]. In the middle of the chronic steady phase, the amount of IFN-γ secreted by stimulated splenocytes of BALB/c is reestablished and parallels those of the resistant mice [118]. This difference does not relate to a lower ability of BALB/c to generate IFN-γ because in vitro stimulation of naïve splenocytes later in time generates comparable quantities of this cytokine in both murine strains [118]. Rather, it seems connected to a reduced expression of the IL-12 receptor in BALB/c mice. Since responsiveness to IL-12 is linked to INF-γ production, a temporal suspension of the Th1 response seems to occur in BALB/c at the beginning of the chronic steady phase [114,118]. It is important to note that IFN-γ production appears to be mainly promoted by CD4+ rather than by CD8+ lymphocytes [65,149].

Serum IL-6 peaks at the end of the acute phase and remains relatively high until the first 2 weeks of the chronic steady phase [149]. Moreover, upon stimulation with killed *Brucella*, spleen cells from infected mice still produce significant amounts of IL-6 in the middle of the chronic steady phase [149]. Similarly to the production of IFN-γ, the generation of IL-6 seems to be mainly promoted by CD4+ lymphocytes [17,65,149]. However, while the ex vivo generation of INF-γ only responds to the cognate antigen, the ex vivo generation of IL-6 can also be induced by heterologous antigens such as killed *Listeria*[149]. This is reminiscent of the Mackaness effect, in which the maximum activity against live *Listeria* is expressed by the *Brucella* infected mice at the beginning of the chronic steady state, mainly through activated macrophages directly stimulated by CD4+ cells [119,153]. IL-10, GM-CSF, and IL-4 are barely secreted during most of the chronic steady phase by spleen cells of *Brucella* infected mice challenged ex vivo with cognate antigens [17,65,122,138]. In addition, CD4+ cells stimulated ex vivo with *Brucella* antigens produce close to twenty times less IL-2 than the same cells stimulated with concanavalin A [17]. At the end of this phase, splenocytes may be re-stimulated to produce INF-γ, IL-10, GM-CSF and IL-2 [65]. The significance of this is unclear. Increase of spleen regulatory T cells may be directly involved in the suppression of effector T lymphocytes devoted to cytokine release and to the control of the infection [4,120].

Th1 immunity is also denoted by the distribution of different antibody isotypes against *Brucella* LPS during the chronic steady phase (Figure 5B). While no endogenous production of IL-4 is detected at any time during infection, there is a predominance of IgG3 and IgG2a, with a minimum response of IgG1 at the end of the acute phase and up to the 14^th^ week pi [138]. In addition to the anti-LPS specific response, a polyclonal IgG3 and IgG2a production dependent upon the endogenous IFN-γ has also been observed in *B. abortus* infected mice [143] as well as in mice immunized with killed *Brucella*[156]. Moreover, the IgG3, IgG2a, IgG2b, IgG1 isotype profiles generated against purified *Brucella* LPS are also generated in both euthymic and athymic mice [157]. This suggests that INF-γ influences the IgG isotope synthesis in mice by Th1-dependent mechanisms and also by T-independent responses.

One striking phenomenon is the second wave in the increase of foot-pad thickness observed between the 3^rd^ and 8^th^ week (Figure 6). Although this second wave has been related to type IV delayed hypersensitivity reaction [153], it corresponds most likely to a mixed reaction with intervention of type III hypersensitivity. Indeed, the increasing levels of antibodies and antigen after the 2^nd^ week of infection (Figure 5B) are in agreement with the occurrence of a type III reaction.

### Chronic declining phase

**Figure 10:**
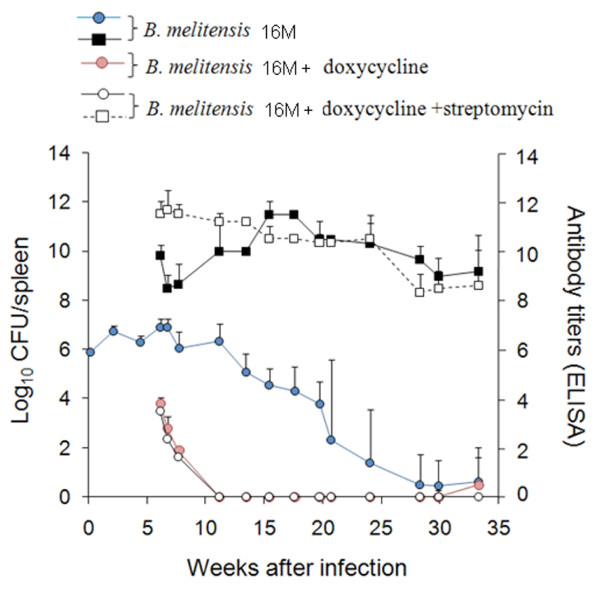
**Antibiotic treatment and antibody response of* B. melitensis* 16 M infected mice.** Blue, pink and white circles represent log_10_ CFU/spleen in the left ordinates axis of the figure. White and black squares represent ELISA values at the right ordinates axis of the figure. Notice that the antibody titers in mice treated with antibiotics remain high over the 34 week period of the assay, in spite of the disappearance of *Brucella* CFU from the spleen. Some of the animals treated only with doxycycline may still harbor bacteria after 34 week period (adapted from [158,159], with permission).

Despite being longest (more than 250 days), the chronic declining phase has been the least studied one [31]. Although there is a declining in DTH [119] and most blood cytokines decrease or become undetectable, the memory response seems fully consolidated because this phase is characterized by a bacterial declining in the target organs and the progressive disappearance of splenomegaly and pathological lesions in liver and spleen [59]. The reduced number of liver granulomas [65] parallels the decrease of IFN-γ. The spleen diminishes in size but it seldom reaches a normal dimension, even at the latest times of infection (Figure 2A), possibly because of the persistence of small numbers of *Brucella* in several organs, mainly in lymph nodes. Nevertheless, *Brucella* is rarely isolated from the blood. Spleen cells maintain their ability to be restimulated by *Brucella* antigens at least for 5 months after infection (Figure 6B) [152], demonstrating long lasting immunological memory against brucellosis [36]. Moreover, antigen stimulated spleen cells produce GM-CSF, IFN-γ and some IL-10, but not IL-4, 6 to 12 months pi [65]. The secretion of INF-γ at these later times is related to CD4+ T cells, since functional blockade of CD4+ T cells by the addition of CD4 + −specific antibody abrogates the cytokine response [65]. The antibody levels stay high throughout the infection period (Figure 10). The sequelae observed in chronic brucellosis in humans and cetaceans [77,129] have not been reported in mice.

## Vaccination

The availability of successful live *Brucella* vaccines for over 75 years (since the discovery of S19) is outstanding [6] because such early developments are exceptions rather than a rule in the field of attenuated vaccines. In spite of their success, the available anti-*Brucella* vaccines are not perfect [22] and their use is restricted to bovines, goats and sheep [16]. There is an increasing interest in developing vaccines for humans and animals other than those domestic ruminants, including wildlife, none of which are covered by the available vaccines [87,128].

Routine testing of *Brucella* vaccines in the natural hosts is precluded due to economical and practical difficulties. Therefore, the mouse model has been extensively used [16,22,33,36,68]. There is, however, substantial anarchy in the protocols used. The literature is plenty of experiments in which the protective efficacy is assessed by comparing the levels of infection of vaccinated and non-vaccinated mice as the only control. By this criterion alone, practically all live strains (no matter whether they are partially or fully attenuated) or even killed *Brucella* provide significant levels of protection [22,29,35,36,68,160]. Moreover, immunization with *Brucella* LPS, outer membrane proteins, bacterial extracts or even with phylogenetically related bacteria (e.g. *Ochrobactrum*) may reduce infection with respect to unvaccinated mice [161-165]. The fact that both cell mediated immunity and antibodies protect mice against brucellosis [107,166] may explain in part why a broad collection of immunogens have such a protective action. A second factor to consider is the Mackaness effect: an “unspecific” activation of the immune system that can protect against *Brucella* challenge within certain periods [153]. Other facts are the particularities of the immune system and short life span of mice, as well as the experimental time intervals used.

The protective efficacy greatly depends on the *Brucella* virulence and challenge dose. Thus, a considerable number of studies has been dedicated to standardize the mouse model for vaccine testing [23,24,32,75,97,167,168]. As a result, a standardized protocol for controlling the quality of *B. abortus* S19 and *B. melitensis* Rev 1 vaccines has been accepted by the World Organization for Animal Health (OIE) [16]. The premise of this model is the fact that these efficient smooth vaccines retain a significant degree of persistence in the host that renders them highly immunogenic and protective [23,24,75]. Since a direct relationship between attenuation and protective efficacy cannot be unambiguously established, this model requires the simultaneous determination of two parameters [32-34,167,168]. The first parameter is the Residual Virulence expressed as RT50 (see above). The second parameter is the Immunogenicity, defined with respect to the ability of the vaccinated mice to control the number of bacteria in the spleen after a standardized challenge [32] (see below). This pa-rameter has to be analyzed at precise intervals after vaccination and is expressed as the mean CFU number of the challenge strain in the spleens. These two parameters do not stand alone, and each vaccine candidate or new vaccine batch should be compared with standard Rev1 or S19 reference strains of known origin and performance. In the case of Rev1 and S19, doses of 10^8^ CFU/mouse injected by s.c. route are used to define the RT50. The same statistical approach may be applied to determine the RT50 of other *Brucella* strains, no matter whether they are virulent, attenuated or non-virulent. However, for non-virulent brucellae, like the VirB or BvrS/BvrR mutants that persist just for a short period, the intervals for spleen culture have to be shortened (e.g. every 3 days) [37]. On the contrary, for assessing the RT50 of virulent strains like *B. melitensis* H38, *B. melitensis* 16 M, *B. abortus* 544, *B. abortus* 2308, and *B. suis* 1330, the inoculum should be much lower (e.g. 10^4^ CFU/mouse). This is so because the virulent *Brucella* strains inoculated at larger doses (e.g. > 10^5^/mouse) persist longer than 30 weeks in the spleen and the RT50 calculation becomes unpractical [31]. The recommended mice are outbred CD-1 or inbred BALB/c females, with body weights ranging between 20–23 grams. Although these mouse strains seem equally susceptible to *Brucella*, the optimal challenge dose for immunogenicity assessment may vary according to the age of the animals, whose body weights relate to their particular growth rates (8–10 weeks of age for BALB/c strain and 4–6 weeks for CD-1.

The original OIE protocol was expensive and cumbersome. The graphical statistical procedures initially proposed for determining the RT50 values had some mathematical complexity and other unpractical inconveniencies that limited its use [33]. For a friendlier statistical tool that facilitates the RT50 (details see Table 3). Two criteria must be met for an adequate RT50 estimation: *i*) four time points must be analyzed for presence of bacteria in the spleens; and *ii*) these time points should be such that the first and the last yield an accumulated percentage of cured mice ≤ 16 % and ≥ 84 %, respectively. Statistical comparisons should be performed exclusively between RT50 values obtained with the same protocol and, ideally, in the same experiment. Alternatively, an indirect or relative estimation of the Residual Virulence of a given strain could be obtained assessing the splenic growth curves described above. In this case, at least one of the reference Rev1 or S19 strains has to be included in the study [22].

**Table 3:** **Problems when performing experiments with*Brucella*in the mouse model and general recommendations**

Experimental variables	Problems	Recommendations
Mouse breed	- Different susceptibility to *Brucella* infections. - Heterogeneity of infection.	- Eight to ten weeks old (20 g) female BALB/c.- For BALB/c, *n* = 5 gives homogeneous spleen counts. For outbred breeds (e.g. CD1) the number per group should be increased (*n* ≥ 6) and the body weight factor taken into account (see Vaccination). For the recommended weight at the Mouse Phenome Database [169].
Target organ	- Inconsistent infection in some organs.	- Count CFU in spleens (consistently colonized in infected animals; longer persistence than in liver or other organs) after determining the individual organ weight.
*Brucella* wild-type challenging strain and virulence controls	- Attenuation by inappropriate storage and/or handling. - Species, biovar and reference strain differences.	- Use only reference strains (*B. abortus* 2308 or 544*, B. melitensis* 16 M or H38, *B. ovis* PA, *B. suis* 1330). Aliquots should be maintained in cryoprotective solution at–80°C (or below), or freeze-dried at 4°C.- Stocks seeded on agar plates and then cultured only once on new plates to obtain final inoculi.- Plates checked for contaminants and freed from condensation or syneresis water.- Make sure that reference strains reproduce typical virulence patterns (see The Brucella strains: replication patterns and related effects)
Attenuated *Brucella* strains and virulence control	- Over-attenuation by inappropriate storage and/or handling. - Inappropriate infectious dose. - Lack of appropriate controls. - Competing events in superinfection protocols	- See above for storage and inoculum preparation.- Typical multiplication (acute phase) and persistence (chronic phase) patterns in spleens should be assessed.- Use adequate virulent controls (see above, Wild-type strains). In genetic manipulation experiments, consider appropriateness of complemented strains and controls for unrelated attenuation cause by in vitro manipulations.- Avoid using protocols in which mixtures of virulent and attenuated *Brucella* strains are used as an attempt to determine the relative virulence of the former in relation to the latter bacteria.
*Brucella* infectious dose and route	- Not optimized for the purpose of the experiment. - Dose not adequate to the route. - Animal handling during inoculation.Intrinsic problems in some routes.	- Use PBS pH 6.85 for preparing the inoculum.- In a preliminary dose–response assay, determine the optimal dose/route (see Route of the infection) for each *Brucella* species, biovar and reference strain (e.g. *B. abortus* 2308 vs. 544 or *B. melitensis* 16 M vs. H38) and also for each mouse breed. For outbred mice, doses must also be adapted to the body weight/age (Physiopathology).- The intraperitoneal route is recommended for most purposes. To avoid intra-intestinal inoculation, displace the intestines and inoculate in the lateral of the abdomen (not in the *linea alba*). Intravenous inoculation in tail is exceedingly difficult and poses repeatability problems, particularly in small breeds. Oral and aerosol routes are not recommended (see Route of the infection).
Vaccination/attenuated strains dose	- Inappropriate dose and route. - Absence of controls	- For classical smooth vaccines, follow the OIE protocol (1 × 10^5^ CFU/subcutaneously).- To evaluate *B. melitensis* and *B. abortus* vaccines, always include Rev1 or S19 reference strain controls, respectively.
Time intervals for virulence studies	Not meaningful.	- For screening, analyze two times corresponding to the multiplication phase and the persistence (e.g. 2 and 8 weeks post-infection). For definite results, test four times (e.g. 2, 4, 6, and 8 weeks post-infection).
Assessment of vaccine efficacy	- Challenge strain. - Time intervals. - Differentiation of vaccine and challenge strains.	- Use fully virulent reference strains and a control reference vaccine strain (see above).- Challenge 4 weeks after vaccination (see Vaccination).- Whenever possible, the challenge strain should carry identifiable marker(s).
CFU determination	- Limit of detection not optimized. - Expression of results (CFU/organ vs. CFU/weight)	- Homogenize the organ in 1:9 (weight:volume) PBS and plate 100 μL by triplicate of each dilution (limit of detection of this method = 3.3 CFU/mL of dilution, corresponding to less than 5 CFU/spleen)- Express the results as log CFU/organ and report spleen weights separately (inflammation varies depending on the *Brucella* strain and among mouse strains).
Evaluation of immune response	- Presence of antigens and bacteria when performing ex vivo experiments. - Lack of correlation between transcripts and protein immune mediators - Lack of sensitivity- Lack of specificity - Inappropriate plotting of data	- Procure APC from non-infected mice to avoid dragging antigen or bacteria. Whenever possible perform direct assays (e.g. flow cytometry, microscopy, cell protein extraction, and serum detection).- Contrast the results obtained with indirect methods with those generated by direct methods.- Use times of maximum expression of cell types or immune mediators.- Consider the “Mackaness effect”.- Include a saturating control that could reveal the real magnitude of the response. Whenever possible, avoid expressing data in relative numbers or “fold responses” and procure the inclusion of absolute values.
Assessment of depletion of cells and immune factors	- Inefficient depletion	- Check antibody concentration, reactivity, dose and time intervals of administration. Consider that depletion seldom last more than 8 days due to neutralization by generation of anti-antibodies
Statistical analysis	- Inappropriate normalization and statistical tests - Outlier values	- Transform logarithmically the individual number of CFU/spleen, calculate the mean Log_10_ CFU/spleen, and compare means by the Fisher’s Protected Least Significant Differences test (PLSD), using a maximum of 4 groups per comparison (including reference or wild-type strain and, for protection studies, both the reference vaccine and placebo control groups). The RT50 calculations should be performed in the freely available statistical program at [170]. - If controls do not give expected values, the assay should be discarded (do not remove outliers).

For Immunogenicity assessment, mice are injected s.c. with the reference vaccine at doses of 10^5^ CFU/mouse. Thirty days after vaccination, mice are challenged by the i.p. route with the standard challenge dose (2 × 10^5^ CFU) of CO_2_-dependent *B. abortus* 544. Two weeks later, the CFU of the challenge strain are counted in the spleens using differential growth conditions for identifying vaccine and challenge colonies^a^. Under these conditions, immunogenicity is expressed as the mean ± SD of log_10_ (X/log_10_X), where X is the number of CFU in each individual spleen. The mean log_10_ (X/log_10_X) obtained in the testing vaccine group should be compared with that obtained in the vaccinated and unvaccinated control groups, preferentially by ANOVA followed by the Fisher's Protected Least Significant Differences tests.

Since protection against *Brucella* infection (at least in the mouse model) is genus-specific and not species-specific [75,168], *B. abortus* 544 has been recommended as the standard challenge strain, even when *B. melitensis* Rev1 is used as vaccine. In spite of this, and whenever possible, a homologous challenge strain should be used. Accordingly, species-specific reference virulent strains such as *B. melitensis* H38 or 16 M for anti-*B. melitensis* vaccines, *B. abortus* 544 or 2308 for anti-*B. abortus* vaccines and *B. suis* 1330 for anti-*B. suis* vaccines have been used as a challenge in some works [22,72,75,81].

In all cases, the vaccine must also be easily distinguishable from the challenge strain. Live vaccines may survive longer than the time of challenge, and even become reactivated [23]. As consequence, they may be present when the number of CFU of the challenge strain in the spleen is estimated. To distinguish the vaccine and the challenge strains on the culture plates, markers like erythritol sensitivity (e.g. S19), CO_2_ dependence (e.g. *B. abortus* 544), antibiotic resistance (e.g. *B. melitensis* Rev1) or antibiotic sensitivity (e. g. *B. abortus* 2308 nalidixic acid sensitive) have been used [22,75,87,171,172]. As an alternative, the vaccination-challenge interval can be extended to ensure the complete elimination of the vaccine from the spleen [23,24]. Following this method, challenge with *B. abortus* 2308 or *B. melitensis* H38 in BALB/c mice generally render more reproducible results than those obtained with *B. abortus* 544 or *B. melitensis* 16 M [22,35,68,172]. One reason for this is that the two former strains give a broader range of values between controls inoculated with the placebo or the reference vaccine. The replication of the challenge strain in mice vaccinated with a reference vaccine (S19 or Rev1) is lower than in unvaccinated mice during the first 2 weeks and, therefore, the CFU/spleen reach very different values [23,24]. After the 4^th^ week, the CFU values in vaccinated and unvaccinated mice decrease in parallel usually maintaining a difference of two to three logarithms. Thus, vaccine efficacy is generally analyzed 2 weeks after challenge, when the differences between the reference-vaccinated and unvaccinated controls are already at a maximum, and longer times do not offer a better discrimination span with respect to protection.

Different statistical procedures for evaluating vaccine efficacy are used. The Fisher's Protected Least Significant Differences or the Bonferroni's tests (depending on the number of groups compared) usually give statistically weighted (i.e. equilibrium between alpha and beta errors) results. In this, both reference-vaccinated and unvaccinated controls act respectively as upper and lower reference limits of protection in the ANOVA test. As indicated above, the level of protection is expressed as log_10_(X/log_10_X) in the reference method. Reportedly, this transformation allows better data normalization than log_10_X when this value is below 1.58 [173] but in the authors’ experience it is not necessary in most cases^b^.

**Figure 11:**
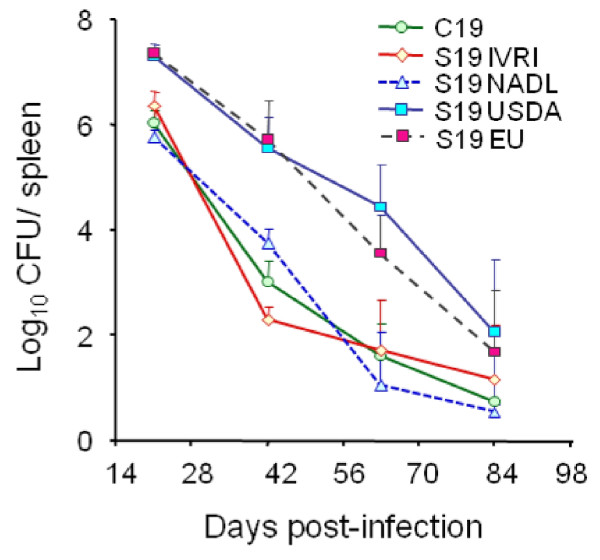
**Splenic growth curves of BALB/c mice infected with several* B. abortus* S19 vaccine strains, from different sources.** Mice were intraperitoneally inoculated with 10^5^ CFU/mouse of the corresponding strain and the number of bacteria estimated in the spleens at different times pi (adapted from [34], with permission).

It is necessary to emphasize that new vaccine candidates as well as stocks or batches of commercial S19 and Rev1 should be contrasted with well-standardized *Brucella* reference strains applying always the appropriate statistical methods to measure both virulence and efficacy [33]. Variations in S19 virulence (Figure 11) and Rev1 efficacy [32] among different stocks have been detected using this methodology.

## Superinfection and antigen therapy

One common practice in some countries has been the revaccination or vaccination of infected animals [174]. Since abortion tends to stop or decrease after mass vaccination with smooth vaccines, it has been hypothesized that vaccination of infected animals may have some therapeutic effect. In addition, vaccinated animals cohabiting with infected flocks may be naturally challenged and become infected with *Brucella* field strains [175]. Moreover, “antigen therapy” has been used to treat human brucellosis and claimed to be effective in improving the clinical status of brucellosis infected patients [3,9,176]. However, these practices are of dubious value and most of them have been abandoned because of the undesirable side effects. In this regard, the mouse model has revealed significant flaws in the claims made on the usefulness of these practices in human medicine.

Treatments with either live brucellae or subcellular bacterial antigens during the acute or chronic phases do not modify the course of *Brucella* infections in mice [29,81,83]. This resembles what happens in the natural hosts [175]. It seems that, whereas the immune response has reached a “saturating level” in the acute and steady chronic phases (related to an extensive *Brucella* reticuloendothelial system colonization), the declining chronic phase resembles an immunosuppression state, an event also observed in chronic brucellosis [4,177]. This last phenomenon may be associated to a progressive decrease of macrophage activation [153,177] as well as to immune regulatory mechanisms [4,65].

The evolution of brucellosis after superinfection as a treatment in mice is related to the virulence of the strains involved. Macrophage reactivation has been reported after inoculating *Brucella* antigens in *B. abortus* S19 infected animals [178]. Similarly, *B. abortus* S19 reinoculation in previously S19 infected mice results in a regression of the primary infection [153]. Nevertheless, this apparent therapeutic effect is questionable. Indeed, a transient reactivation of the attenuated *Brucella* strains may occur. In S19 vaccinated mice, the vaccine strain may become reactivated and increase in numbers after inoculation with virulent *Brucella*, brucellin or LPS [23]. This suggests that S19 does not “saturate” the reticuloendothelial system as it is the case of virulent strains. Moreover, mice infected with attenuated brucellae are less resistant to challenge with virulent strains than animals already infected with virulent *Brucella*[179]. These competing events are not trivial, mainly in protocols in which mixtures of virulent and attenuated *Brucella* strains have been used as an attempt to determine the relative virulence of the former in relation to the latter bacteria [91].

## Passive transfer and immunomodulation

Passive transfer of antibodies and cells, and treatment with cytokines and immune enhancers are widely used methods to investigate the immune responses during murine brucellosis (Table 2). These procedures may be divided into five groups: *i*) passive transfer of antibodies; *ii*) passive transfer of immune cells; *iii*) treatment with cytokines or immunomodulators; *iv*) antibody depletion of cytokines; and *v*) antibody depletion of immune cells.

Passive transfer of homologous or heterologous polyclonal antisera from infected or immunized animals (syngenic, allogenic or xenogenic) protects mice against *Brucella* challenge [84,98,106,107]. Using monoclonal antibodies (Mabs), it has been established that the most relevant targets are epitopes of N-formylperosamine sugars of the O chain of the LPS and NH polysaccharides [1] as well as some Omps like Omp31, Omp25 and Omp16 (Table 2). No correlation between the protection mediated by passive antibody transfer and the immunoglobulin isotype has been recorded. Strikingly, the efficient clearance of *Brucella* observed in the spleens of B cell deficient mice (Table 1) is not reversed or improved by passive administration of immune serum [55]. This suggests that the control of *Brucella* infection is also dependent on some B cell effectors not necessarily related to the presence of antibodies.

Passive transfer of immune splenocytes or purified populations of CD4+ and CD8+ T cells obtained after 4 weeks of infection [98,112] protect mice against brucellosis (Table 2). As expected, a higher protection is achieved when both immune sera and cells are transferred simultaneously [107]. Similarly, passive transfer of xenogenic macrophages together with autologous Tγδ cells protects mice against *Brucella* replication; however, passive transfer of xonogenic macrophages alone or macrophages with autologous unprimed T cells do not (Table 2). In addition, the administration of cytokines generated during the Th1 response (e.g. INF-γ and IL-12) or the injection of immunostimulants (e.g. poly-A:U or indomethacin), promote the elimination of *Brucella* in mice. Indomethacin (used to stop suppressive actions mediated by the secretion of cyclooxygenase-dependent prostaglandins) does not prevent the decline in *Brucella*-induced INF-γ production [113]. Similarly, IL-1α (involved in the early activation of macrophages and recruitment of cells) induces protection during the acute and chronic phases of brucellosis when administered before infection [28]. In contrast, general immunossupresive agents such as corticosteroids, enhance *Brucella* proliferation and avoid inflammation in the target organs [3].

Depletion of immune cells has revealed significant but controversial results (Table 2). For example, antibody mediated depletion of B and NK cells barely influences the outcome of *Brucella* infection. In contrast, removal of CD8+ favors the invasion and increase of *Brucella* numbers in the spleen of mice. Noticeably, antibody mediated depletion of PMNs, CD4+ or CD25+ T cells favors the elimination of *Brucella* from the target organs in mice, suggesting some regulatory events. Indeed, these cells may exert some suppressive regulatory action on macrophages and dendritic cells, and perhaps on T-cytotoxic lymphocytes [4], all cells that constitute primary defenses against brucellosis.

Antibody mediated depletion of INF-γ, IL-12 and TNF-α promotes *Brucella* replication in mice. In contrast, depletion of regulatory IL-10 or IL-4 favors *Brucella* elimination (Table 2). This last event may be the result of balancing the immune response towards Th1, and therefore, favoring the efficient elimination of brucellae by cell mediated immunity. Depletion of CD8+ T cells results in a significant increase in *Brucella* numbers, which is associated to macrophage increase in the spleen. As expected, depletion of Tγδ cells, participating in innate immunity also favors *Brucella* replication. The role of TNF-α may depend upon the presence of INF-γ early in the infection, since when TNF-α is neutralized in INF-γ deficient mice there is an increase of macrophages, NK cells and neutrophils in the spleens [57]. These effects are in keeping with the preferential Th1 immune response during brucellosis, as well as with the participation of TNF-α in activating phagocytic cells, mainly during the acute and early chronic phases.

## Antibiotic treatment

The mouse model has been successfully used to evaluate antibiotic doses, delivery, and efficacy, as well as for studying the course of brucellosis and antibody response during and after antibiotic treatments [159,180,181]. As in the human disease, murine brucellosis is better treated by a combination of doxycycline and aminoglycosides (streptomycin or gentamicin), or rifampicin [159,182,183]. While doxycycline also exerts its killing action inside cells, the second group preferentially acts extracellularly. Rifampicin is capable of reducing the *Brucella* loads in the spleen of mice, since it can penetrate inside leukocyte vacuoles. Consequently, it has better intracellular activity than aminoglycosides [158,183]. The effectiveness of rifampicin or gentamicin combined with doxycycline against streptomycin-resistant *B. melitensis* Rev1 has been demonstrated both in humans and mice [184,185]. However, treatments with a combination of gentamicin-doxycycline seem to be the most efficient against this vaccine strain.

The combination of doxycycline (50 mg/kg body weight/12 h for 45 days, orally), and streptomycin (i.p., 10–20 mg/kg body weight/l2 h, for 14 days) is the most efficient treatment against brucellosis in mice. This regime, which is very similar to that given to humans, does not cause relapses in mice, as far as 7 months after antibiotic treatment [159]. Accordingly, 13 days after treatment, the bacterial loads in the spleen are reduced more than three logarithms with respect to untreated mice, and bacteria are not detected in the spleen after 47 days of treatment (Figure 10). This combined regime is superior to single treatments. Indeed, regimes using only doxycycline do not completely eliminate the bacteria and may cause relapses after the 30^th^ week of infection (Figure 10). Efforts to find substitutes for doxycycline and alternative tetracyclines for brucellosis treatment have failed. Despite the good brucellicidal action in vitro, experiments in mice using fluoroquinolones (moxifloxacin, gatifloxacin, ciprofloxacin and levofloxacin) or macrolides (erythromycin, dirithromycin or azithromycin), have been unsuccessful [180,181,186-188].

Various protocols to evaluate intracellular delivery of antibiotics inside the *Brucella* replication vacuole have been tested in mice. Gentamicin containing microspheres, obtained by spray drying, reduced significantly the splenic infection in mice after i.p. or i.v. administration [188]. However, some mice died of pulmonary embolism due to aggregation of the particles. Attempts to solve this were done by including gentamicin within polymeric nanoparticles made out of D,L-lactide-coglycolide [189]. In this model, gentamicin-containing microspheres administrated i.v. reduced but not eliminated the burden of *B. melitensis* infection. Interestingly, the microsphere body distribution was similar to that followed by *Brucella* organisms, being spleen and liver the main target organs. An alternative approach to improve antibiotic treatment has been the inclusion of streptomycin and doxycycline into macromolecular nanoplexes [190]. Intravenous administration of two doses reduced the number of *B. melitensis* 16 M in spleens and livers of mice, and seemed more effective than free drugs. Pharmacokinetics of these nanoparticles containing antibiotics has not been studied.

Decline of antibody titers against LPS and proteins after antibiotic treatment in human patients frequently corresponds with successful elimination of the *Brucella*[182]. In contrast, titers of antibodies against *Brucella* LPS have a tendency to remain elevated in treated mice (Figure 10). This is evident with high challenge doses (e.g. >10^6^*B. melitensis* CFU/mouse) and with antibiotic regimes (e.g. doxycycline or doxycycline-streptomycin) given orally after the 3^rd^ week of infection [159]. The difference between mice and humans may be due to the shorter life span of the former and/or the ability of anti-LPS antibodies to protect mice against brucellosis (Table 2). However, when treatment starts during the 1^st^ week of infection and the challenge dose is lower (<10^4^*B. melitensis* CFU/mouse), the antibody response against *Brucella* cytoplasmic proteins decreases to undetectable levels in mice [191]. This parallels the low or lack of anti-protein responses in human patients receiving early antibiotic treatment [192].

## Concluding remarks

There are several differences and similarities between experimental brucellosis in mice and the disease in the natural hosts and in humans. For instance, mice are quite resistant to *Brucella* infection but, in contrast to natural hosts, do not seem to shed *Brucella* significantly and the infection seems to be contained. Therefore, horizontal transmission does not seem important in experimental murine brucellosis. In non-treated human patients or in dolphins, brucellosis may become chronic, causing cardiopathies, extensive bone lesions and neurobrucellosis, as well as other severe pathologies [9,77,129]. Strikingly, all these syndromes are seldom recorded in infected mice when using current protocols and adequate bacterial doses (e.g. large doses may saturate the organs and overcome the immune response). In mice, the liver is the shock organ and the inflammatory immune response in the spleen protects the liver from massive *Brucella* invasion [14,15]. Similarly, humans also display splenomegaly and during chronic brucellosis the liver becomes one of the sites for *Brucella* replication [3,9]. In contrast, prominent liver granuloma formation and large splenomegaly have seldom been reported in ruminants [130]. While active spleen hematopoiesis is a conspicuous feature in murine brucellosis, it is a very rare event in humans and ruminants [21]. A common feature of murine, human and domestic animal brucellosis is the absence of endotoxic symptoms at the onset of the infection, a phenomenon related to the stealthy strategy of *Brucella*[4,48].

As in other mammals, the mouse giant mononuclear placental trophoblasts are also target cells for *Brucella*. The invasion of placenta occurs at specific periods in experimentally infected mice and natural hosts but with different clinical consequences. In fact, *Brucella* infections occurring later in pregnancy (i.e. after the last one-third of pregnancy) or close to delivery, induce less abortions in both natural hosts and mice [135]. However, there are fundamental differences between placental infections in mice and in natural hosts. Mice are quite resistant to *Brucella* induced abortion and abortion is linked to a particular immune response within a narrow window of the gestation period in which the placenta is effectively colonized by the invading *Brucella*[135]. In contrast, in bovines and small ruminants, abortion generally occurs during the last one-third of pregnancy, irrespectively of whether they were infected months or years before [8,130,193]. This difference between mice and ruminants may be related not only to the nature of trophoblastic cells but also to placental structure [194]. For instance, in ungulates there is no transplacentary transfer of antibodies while in rodents there is a significant transmission of antibodies from the mother to the fetus. This is relevant since the antibody response in mice against *Brucella* could protect the fetuses. Regarding the reproductive organs of male mice, *Brucella* colonize the testes very early after experimental infection [76] with invasion of the epididymis [128], a phenomenon that resembles brucellosis in human, bovine, ovine and caprine males.

The understanding of immune mechanisms during brucellosis has been a significant contribution of the mouse model. The furtive strategy of *Brucella* to overcome the innate immunity at the onset of the infection, the role of INF-γ and the Th1 responses in controlling brucellosis during the rise of adaptive immunity, have been partially elucidated using the mouse model [4,48,66,103,125]. There are, however, significant discrepancies in both innate and adaptive immunity mechanisms between mice, humans and ruminants [20,21,195,196]. For instance, mouse macrophages seem to be more bactericidal and less permissive than human macrophages [66,197]. The mouse resistance to *Brucella* may be also related to a higher proportion of lymphocytes in relation to other cells such a neutrophils. The murine C'2 and C'3 complement activities are quite low, and the serum contains a potent inhibitor of lytic activity precluding this function in mice. The properties of immunoglobulin isotypes are also different in mice, bovines and humans. Other significant variations are the absence of defensins in mouse leukocytes, different subsets of TLR, inducible NO synthase, the NK inhibitory receptor families Ly49 and KIR, FcR, the B cell (BLNK, Btk, and λ5), T cell (ZAP70 and common γ-chain) signaling pathway components, Thy-1, γδT cells, cytokines and cytokine receptors, Th1/Th2 differentiation, costimulatory molecule expression and function, antigen-presenting function of endothelial cells, chemokines and chemokine receptor expression and the absence of granulysin in murine Tc lymphocytes [21].

It is intriguing that various acquired or innate immune deficiencies do not seem to alter the outcome of *Brucella* infection in mice (Tables 1 and 2). For instance, neutrophil depletion and B cells immune deficiency seem to favor the elimination of *Brucella* from the spleens of the corresponding treated or knockout mice more readily than what it happens in the wild type. This suggests that complex immune regulatory and compensatory events may take place during *Brucella* infection in mice [4]. It has been proposed that the augmented bacterial clearance in B-cell deficient mutant mice corresponds to an increase in IFN-γ-producing T cells and a reduction in IL-10-producing cells [55]. Similarly, in B cell deficient *jh* knockout mice, bacterial clearance seems also to be dependent on IFN-γ production but inversely related to the levels of TGF-β at early stages of infection.

Some confusion results from the different methods used to evaluate the immune responses in mice. The most reliable are the classical methods that consist in: i) determining the DTH, the amount of antibodies and cytokines in sera by immunochemical assays; and ii) the number and type of cells in blood and organ by microscopy or cytometry. In spite of their relative low sensitivity, these methods are specific and render consistent results. The interpretation of the immune response by ex vivo indirect assays, in which cell proliferation or cytokines are measured in cell cultures exposed to antigens, is not straightforward, particularly when cytokines are indirectly determined by detecting transcripts. Among other sources of variation, the immune cells harbor *Brucella* antigens and frequently live bacteria depending upon the time at which cells are taken from the infected mice (e.g. 7 to 60 days). This fact is rarely mentioned or taken into consideration in experimental works. Similarly, the estimated transcripts seldom parallel the quantitative measurement of proteins [195]. Therefore, it is recommended to contrast the results obtained by indirect procedures with those generated by direct methods.

A significant number of reports present replication and persistence patterns of reference or type *Brucella* strain that do not reproduce those established in mice. Generally, this means that the *Brucella* type strain used has become attenuated, and this is important source of misunderstanding, mainly in pathogenicity and virulence studies. Other source of confusion corresponds to the manner in which data are expressed or presented. It is common to notice published information schematically plotted to display “significant” conspicuous values (e. g. bars or peaks) without including a full virulent positive control administered at the optimal dose that could reveal the real magnitude of the response. Taking into account that ordinate axis can represent different scales the risk is that the plotted values may appear significant, when no adequate saturating control (inducing the maximum response) is included. Similarly, in other type of experiments, it is necessary to contrast the results obtained with *Brucella* with those of unrelated infections, mitogens or immunostimulants (e.g. *Listeria, Salmonella*, concanavaline A or LPS) that generate well known saturating responses.

Few live vaccines are able to confer adequate protection in domestic ruminants, and subcellular vaccines or killed bacterins are not protective enough in the natural hosts. This is in clear contrast with many experimental results in mice. Nevertheless, under well-standardized conditions the mouse model is a useful tool for screening vaccines, even though one vaccine that performs efficiently in mice may not work in the natural host. The opposite, however, has not been reported: vaccines that protect poorly in mice are useless in the natural hosts [85,198,199]. In any case, for vaccine evaluation it is always necessary to use controls vaccinated with the reference vaccine strains and to apply the correct statistical tests. A summary of the general problems and recommendations when performing experiments with *Brucella* in the mouse model is presented in Table 3.

*Mus musculus* seems to be the second most abundant and disperse mammalian species in the world, after *Homo sapiens.* This rodent diverged from ungulates and primates about 90 million years ago [200], probably at the time when *Brucella* speciation occurred in various animal hosts [201]. Although for many decades *Brucella* organisms have been isolated in some species of Muroidea, [202,203], *M. musculus* has not been found to be a reservoir of *Brucella*. This is remarkable, taken into consideration that “house mice” have shared habitat and food with humans and domestic animals for millennia, mainly after the domestication of crops and ruminants [204]. Thus, whereas it is clear that *M. musculus* constitutes a valuable non-natural host for brucellosis studies, the advantages and limitations of the model should be properly understood within the right experimental context.

## Endnotes

^a^For example, for *B. abortus* 544 and S19 differentiation, incubate under a CO_2_ atmosphere (where both strains grow) and under normal atmosphere (where only S19 grows) and subtract the values obtained for the same spleen dilution.

^b^For a practical example, see (Additional file 1: Table S1).

## Competing interests

The authors declare that they have no competing interests.

## Authors’ contributions

EM and MJG drafted the manuscript, figures and tables. IM, JMB and JPG helped to draft the manuscript and were involved in revising the manuscript critically for relevant intellectual content. All authors have read and approved the manuscript.

## Supplementary Material

Additional file 1 **Table S1.** Recommended method for calculating the level of spleen infections in mice. Click here for file
