# Supplementary material for: What have we learned from brucellosis in the mouse model?
Source: Vet Res. 2012 Apr 13;43(1):29. doi: 10.1186/1297-9716-43-29 (PMC3410789; doi:10.1186/1297-9716-43-29)
Supplement: Additional file 1 — Table S1. Recommended method for calculating the level of spleen infections in mice. [file 1297-9716-43-29-S1.doc]

**Table S1: Recommended method for calculating the level of spleen infections in mice** a

|  | | **Framework table:** | | | | | | | | | | | | |
| --- | --- | --- | --- | --- | --- | --- | --- | --- | --- | --- | --- | --- | --- | --- |
|  | | **A** | **B** | **C** | **D** | **E** | **F** | **G** | **H** | **I** | **J** | **K** | **L** | **M** |
| **1** | | **Mouse identification** | **Bag weight (grams)^b^** | | **SPLEEN WEIGHT** | **First 1:10 dilution** | Number of CFU isolated in agar plates | | | | **MATHEMATICAL TRANSFORMATION** | | | |
| **2** | |  | **Bag containing the spleen** | **Bag empty** | **(grams)** | **PBS volume (mL)** | **in a volume of:^c^** | | | **Dilution^d^** | **Mean CFU** | **CFU/mL** | **CFU/spleen** | **log_10_ CFU/spleen** |
| **3** | | **Group** |  |  |  |  | **100 μL** | **100 μL** | **100 μL** |  | **in 100 μL** |  |  |  |
| **4** | | **1** |  |  | =(B4-C4) | =(D4*9) |  |  |  |  | =MEAN(F4:H4) | =(I4*J4) | =(K4*(D4+E4)) | =LOG10(L4) |
| **5** | | **2** |  |  | =(B5-C5) | =(D5*9) |  |  |  |  | =MEAN(F5:H5) | =(I5*J5) | =(K5*(D5+E5)) | =LOG10(L5) |
| **6** | | **3** |  |  | =(B6-C6) | =(D6*9) |  |  |  |  | =MEAN(F6:H6) | =(I6*J6) | =(K6*(D6+E6)) | =LOG10(L6) |
| **5** | | **4** |  |  | =(B7-C7) | =(D7*9) |  |  |  |  | =MEAN(F7:H7) | =(I7*J7) | =(K7*(D7+E7)) | =LOG10(L7) |
| **8** | | **5** |  |  | =(B8-C8) | =(D8*9) |  |  |  |  | =MEAN(F8:H8) | =(I8*J8) | =(K8*(D8+E8)) | =LOG10(L8) |
| **9** | |  |  |  | **=MEAN(D4:D8)** |  |  |  |  |  |  |  | **AVER(L4:L8)** | **=MEAN(M4:M8)** |
| **10** | |  |  |  | **=STDV(D4:D8)** |  |  |  |  |  |  |  |  | **=STDV(M4:M8)** |
|  | ^a^ The spleens should be individually: (i) removed, deffated and weighed in a sterile bag; (ii) serially tenfold diluted in PBS; and (iii) plated placing 100 μL of each spleen dilution, in triplicate. | | | | | | | | | | | | | |
|  | For automatic calculations in an excel sheet, only gray shaded cells must be completed, indicating: | | | | | | | | | | | | | |
|  | ^b^ The weight of a sterile bag both empty (column C) and containing the spleen (column B) | | | | | | | | | | | | | |
|  | ^c^ The number of CFU isolated after incubation (37 ºC, 3-5 days) of agar plates previously inoculated with 100 μL by triplicate (columns F, G and H). | | | | | | | | | | | | | |
|  | When CFU is not detected in the first dilution of the spleen (0 CFU), a value of "0.33" (that is the limit of detection in the corresponding 100 mL) should be included in the correspondent window. | | | | | | | | | | | | | |
|  | ^d^ The dilution at which the isolated CFU were counted, indicating so many zeros as the dilution factor (column J). | | | | | | | | | | | | | |
|  | The lower dilution of the spleen (1:10; w:v in PBS) corresponds to a value of "10" and the following dilutions should be expressed as 100, 1000, etc. | | | | | | | | | | | | | |
|  | | **Example:** | | | | | | | | | | | | |
|  | | **A** | **B** | **C** | **D** | **E** | **F** | **G** | **H** | **I** | **J** | **K** | **L** | **M** |
| **1** | | **Mouse identification** | **Bag weight (grams)^b^** | | **SPLEEN WEIGHT** | **First 1:10 dilution** | Number of CFU isolated in agar plates | | | | **MATHEMATICAL TRANSFORMATION** | | | |
| **2** | |  | **Bag containing the spleen** | **Bag empty** | **(grams)** | **PBS volume (mL)** | **in a volume of:** | | | **Dilution** | **Mean CFU** | **CFU/mL** | **CFU/spleen** | **log_10_ CFU/spleen** |
| **3** | | **Group** |  |  |  |  | **100 μL** | **100 μL** | **100 μL** |  | **in 100 μL** |  |  |  |
| **4** | | **1** | 2.17 | 2.10 | 0.07 | 0.63 | 0.33 | 0.33 | 0.33 | 10 | 0.33 | 3.30E+00 | 2.31E+00 | 0.36 |
| **5** | | **2** | 2.20 | 2.05 | 0.15 | 1.35 | 1 | 0.33 | 0.33 | 10 | 0.55 | 5.53E+00 | 8.30E+00 | 0.92 |
| **6** | | **3** | 2.15 | 1.99 | 0.16 | 1.44 | 1 | 3 | 5 | 10 | 3 | 3.00E+01 | 4.80E+01 | 1.68 |
| **5** | | **4** | 2.22 | 2.00 | 0.22 | 1.98 | 20 | 50 | 55 | 100 | 42 | 4.17E+03 | 9.17E+03 | 3.96 |
| **8** | | **5** | 2.32 | 2.07 | 0.25 | 2.25 | 45 | 55 | 60 | 100 | 53 | 5.33E+03 | 1.33E+04 | 4.12 |
| **9** | |  |  |  | **0.17** |  |  |  |  |  |  |  | **4.51E+03** | 2.21 |
| **10** | |  |  |  | **0.07** |  |  |  |  |  |  |  |  | **1.74** |
